# π-Donor/π-Acceptor Interactions for the Encapsulation of Neurotransmitters on Functionalized Polysilicon-Based Microparticles

**DOI:** 10.3390/pharmaceutics12080724

**Published:** 2020-08-01

**Authors:** Sandra Giraldo, María E. Alea-Reyes, David Limón, Asensio González, Marta Duch, José A. Plaza, David Ramos-López, Joaquín de Lapuente, Arántzazu González-Campo, Lluïsa Pérez-García

**Affiliations:** 1 Departament de Farmacologia, Toxicologia i Química Terapèutica, Universitat de Barcelona, Avda. Joan XXIII 27-31, 08028 Barcelona, Spain; sandra.giraldo@ub.edu (S.G.); lyafalea@gmail.com (M.E.A.-R.); davidlimon@ub.edu (D.L.); gazulla53@gmail.com (A.G.); or mlperez@ub.edu (L.P.-G.); 2 Institut de Ciència de Materials de Barcelona (ICMAB-CSIC), Campus UAB, 08193 Bellaterra, Barcelona, Spain; 3 Institut de Nanociència i Nanotecnologia UB (IN2UB), Universitat de Barcelona, Avda. Joan XXIII 27-31, 08028 Barcelona, Spain; 4 Institut de Microelectrónica de Barcelona (IMB-CNM-CSIC), Campus UAB, 08193 Bellaterra, Barcelona, Spain; Marta.duch@imb-cnm.csic.es (M.D.); Joseantonio.plaza@imb-cnm.csic.es (J.A.P.); 5 Parc Científic de Barcelona, Unitat de Toxicologia Experimental i Ecotoxicologia (UTOX-PCB), Baldiri i Reixac 10-12, 08028 Barcelona, Spain; ramos.lopez.david@gmail.com (D.R.-L.); qlapuente@gmail.com (J.d.L.)

**Keywords:** π-donor/π-acceptor complexes, viologens, polysilicon microparticles, drug encapsulation, drug delivery, neurotransmitters

## Abstract

Bipyridinium salts, commonly known as viologens, are π-acceptor molecules that strongly interact with π-donor compounds, such as porphyrins or amino acids, leading their self-assembling. These properties have promoted us to functionalize polysilicon microparticles with bipyridinium salts for the encapsulation and release of π-donor compounds such as catecholamines and indolamines. In this work, the synthesis and characterization of four gemini-type amphiphilic bipyridinium salts (**1·4PF_6_**–**4·4PF_6_**), and their immobilization either non-covalently or covalently on polysilicon surfaces and microparticles have been achieved. More importantly, they act as hosts for the subsequent incorporation of π-donor neurotransmitters such as dopamine, serotonin, adrenaline or noradrenaline. Ultraviolet-visible absorption and fluorescence spectroscopies and high-performance liquid chromatography were used to detect the formation of the complex in solution. The immobilization of bipyridinium salts and neurotransmitter incorporation on polysilicon surfaces was corroborated by contact angle measurements. The reduction in the bipyridinium moiety and the subsequent release of the neurotransmitter was achieved using ascorbic acid, or Vitamin C, as a triggering agent. Quantification of neurotransmitter encapsulated and released from the microparticles was performed using high-performance liquid chromatography. The cytotoxicity and genotoxicity studies of the bipyridinium salt **1·4PF_6_**, which was selected for the non-covalent functionalization of the microparticles, demonstrated its low toxicity in the mouse fibroblast cell line (3T3/NIH), the human liver carcinoma cell line (HepG2) and the human epithelial colorectal adenocarcinoma cell line (Caco-2).

## 1. Introduction

The development of effective methods for drug encapsulation and release are topics of great importance because of the potential enhancement and prolongation of the overall efficiency of the drug as well as their controllable delivery in a target area. Different methodologies for drug encapsulation have been designed, such as: biocompatible complex microcapsules [1], modified nanocarbon materials [2] and mesoporous silica nanoparticles [3]. On the other hand, self-assembly monolayers (SAMs) and microfabrication techniques have been used to endow substrates of several properties to be applied in drug delivery. SAMs are a key tool in surface design of nanolayers for the bioactive coating of biomedical devices [4,5,6]. Different materials have been employed as substrates for the formation of monolayers, but gold and silicon [7,8,9] are the most widely used and studied, because of their biocompatibility and the ease of using thiol and silane chains, respectively, to be linked onto their surfaces to form well-organized monolayers. Moreover, microfabrication techniques allow the preparation of silicon-based devices with a great control of size, shape and monodispersity, properties that are important for biomedical applications and are a drawback of the chemical methods [10,11]. In this regard, microfabrication processes of silicon-based materials have provided polysilicon microparticles (Poly-Si μPs), which can be functionalized with a photosensitizer as singlet oxygen generators [12] or with lectins to obtain encoded Poly-Si μPs for tagging purposes [13,14]. Importantly, different studies have demonstrated their non-cytotoxicity using various cell lines [14,15]. Moreover, multi-material microparticles have been also microfabricated, showing interaction with different cell lines [16] or as innovative silicon-based intracellular sensors [17]. 

On the other hand, stimuli-responsive materials based on supramolecular complexes and their immobilization on surfaces can be highlighted as one of the potentials for drug encapsulation and sensing [18]. In this regard, bipyridinium salts, commonly known as viologens, are π-electron acceptor and redox-responsive compounds, which are able to generate π–π aromatic interactions with electron-rich groups such as amino acids [19] and neurotransmitters [20,21,22]. One of the better studied π-electron acceptor molecule, based on bipyridinium salts and widely employed to form catenanes and rotaxanes, is the cyclobis(paraquat-p-phenylene) (“blue-box”) [23,24,25]. Moreover, viologens have attracted considerable interest not only for stimuli-responsive encapsulation methodologies, but also for other several applications including: building electrochromic devices [26], molecular electronics [27] and redox sensors [28]. Furthermore, the bipyridinium-based salts have also been used to functionalize silver nanoparticles [29], polymeric nanoparticles [30], microspheres [31], mesoporous silica [32], gold [33] and silicon surfaces [33,34], enabling the possibility of immobilizing bipyridinium salts on biocompatible surfaces for further microencapsulation and drug delivery applications. Thus, the combination of biocompatible microfabricated polysilicon-based devices with bipyridinium salts as hosts for a controllable encapsulation and release of π-donor biomolecules can open new opportunities for the delivery of drugs, whose physicochemical properties make their administration difficult. For this reason, in this work we explore the covalent and non-covalent functionalization of Poly-Si μPs with gemini-type amphiphilic bipyridinium salts used as host to assess the encapsulation and release of neurotransmitters, which present low stability in biological environments. 

Neurotransmitters are π-donor biomolecules that play a pivotal role in communication between cells through signal transduction in living organisms [35]. Generally, an imbalance of these neurotransmitter levels could cause psychiatric and neurological disorders such as: depression, schizophrenia, Parkinson’s and Alzheimer’s diseases [36]. Therefore, ensuring adequate levels of neurotransmitters is necessary for preventing and treating undesired brain disorders. In this regard, advances in sensing methods for neurotransmitters’ detection, and the development of carriers for their encapsulation and controllable delivery have been the focus of scientific interest in neuronanomedicine [37,38]. Thus, different carriers for neurotransmitters encapsulation have been described from liposomes [39], polymeric nanoparticles [40], mesoporous silica nanospheres [41] to amphiphilic anionic calix[5]arene micelles [42]. However, the further advancement of new responsive systems for controllable neurotransmitter delivery is still desirable. 

**Figure 1:**
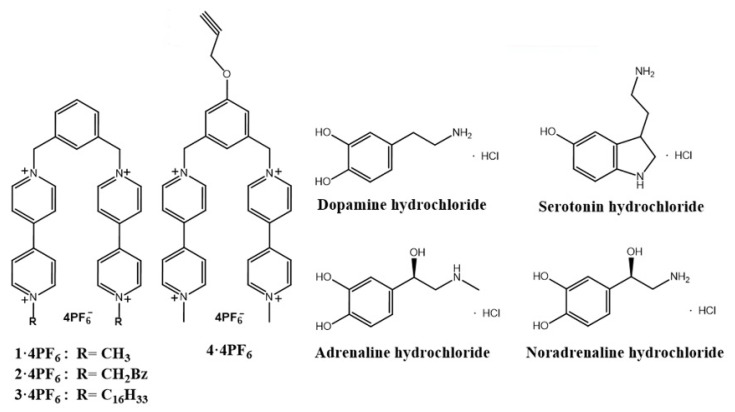
Bipyridinium salts **1**·**4PF_6_**–**4**·**4PF_6_** and neurotransmitters: dopamine hydrochloride (**D**), serotonin hydrochloride (**S**), adrenaline hydrochloride (**A**) and noradrenaline hydrochloride (**NA**).

Herein, this paper describes a new strategy to encapsulate and release neurotransmitters combining silicon-based microfabrication technologies and surface functionalization with bipyridinium salts. With this aim, the synthesis and characterization of four gemini-type amphiphilic bipyridinium salts **1**·**4PF_6_**–**4**·**4PF_6_** is first presented, which act as a host for the subsequent incorporation of the neurotransmitters: Dopamine (**D**), Serotonin (**S**), Adrenaline (**A**) and Noradrenaline (**NA**) (Figure 1). Moreover, a methodology for non-covalent and covalent functionalization of two polysilicon substrates, polysilicon surfaces (wafers) with a pattern of polysilicon microparticles on a thermal silicon oxide layer (Poly-Surfs) and square-shaped polysilicon microparticles (Poly-Si μPs, with **1**·**4PF_6_**–**4**·**4PF_6_** is developed for the further incorporation of the neurotransmitters. This incorporation, favored by π-donor/π-acceptor interactions between the neurotransmitters and the immobilized tetracationic hosts, is characterized by contact angle measurements. Furthermore, the quantification of **A** encapsulated and released using the non-covalent and covalent functionalization of microparticles with **1**·**4PF_6_** and **4**·**4PF_6_**, respectively, is studied using high-performance liquid chromatography (HPLC). Finally, the toxicity of **1·4PF_6_** was determined as model of the bipyridinium salts used, performing cytotoxicity and genotoxicity studies in three different cell lines. 

## 2. Materials and Methods 

### 2.1. Materials

Solvents: Acetonitrile (CH_3_CN), dichloromethane (DCM), dimethyl sulfoxide (DMSO), dimethyl sulfoxide-d_6_ ((CD_3_)_2_SO), *N*,*N*′-dimethylformamide (DMF) and nitromethane (CH_3_NO_2_) were purchased from Merck. Diethyl ether (Et_2_O) was purchased from Carlo Erba and methanol-d_4_ (CD_3_OD) was purchased from Eurisotop.

Reagents: 1-Bromohexadecane, 1,3-bis(bromomethyl)benzene, 4,4′-bipyridine, adrenaline hydrochloride (**A**), 20% ammonia (NH_3_), ammonium acetate (CH_3_COONH_4_), ammonium hexafluorophosphate (NH_4_PF_6_), ascorbic acid, benzyl bromide (BzBr), dopamine hydrochloride (**D**), 30% hydrogen peroxide (H_2_O_2_), iodomethane (MeI), noradrenaline hydrochloride (**NA**) and potassium iodide (KI) were purchased from Merck. 98% Sulfuric acid (H_2_SO_4_) was purchased from Scharlau and serotonin hydrochloride (**S**) was purchased from Alfa Aesar.

Compound **5**, the bipyridinium salt **2**·**4PF_6_**, *N*-methyl-4,4′-bipyridinium iodide and 1,3-dibromomethyl-5-propargyloxy benzene were synthesized following reported procedures [25,43,44,45]. The fabrication process of both polysilicon substrates has previously been reported [12,46]. Neurotransmitter solutions were always freshly prepared and used immediately after their preparation as well as protected from the light.

### 2.2. Instrumentation

Melting points were measured by CTP-MP 300 hot-plate apparatus with ASTM 2C thermometer using crystal capillaries purchased from Afora. ^1^H NMR: Varian Gemini 300 (300 MHz), and Varian Mercury 400 spectrometers (400 MHz) from *Centres Científics i Tecnològics de la Universitat de Barcelona* (CCiT-UB) and a Bruker Avance-400 spectrometer (400 MHz) from *Servei de Ressonància Magnètica Nuclear de la Universitat Autònoma de Barcelona* (SeRMN-UAB) service were employed. ^13^C NMR: Varian Mercury 400 (400 MHz) from CCiT-UB and Bruker Avance-400 spectrometer (400 MHz) from SeRMN-UAB were used. NMR spectra were determined in (CD_3_)_2_SO or CD_3_OD and the chemical shifts are expressed in parts per million (ppm) relative to the central peak of the corresponding solvent. Matrix-Assisted Laser Desorption Ionization-Time of Flight Mass Spectrometry (MALDI-TOF-MS) analyses were performed using a Voyager-DE-RP (Applied Biosystem, Framingham, USA) mass spectrometer, and high resolution mass spectra (HRMS) were obtained by Electrospray (ESI) on a LC/MSD-TOF mass spectrometer (Agilent Technologies, Santa Clara, CA, USA) from CCiT-UB. MS analyses were operated in delayed extraction mode using 2,5-dihydroxybenzoic acid (DHB) as matrix. Infrared spectroscopy (FT-IR) spectra were collected on a ThermoNicolet Avatar 320 FT-IR spectrometer (Thermo Fisher Scientific, Waltham, MA, USA) in a range of 4000–400 cm^−1^, in KBr pellets (sample at 1%) and on a Spectrum One spectrophotometer (Perkin Elmer, Waltham, MA, USA) using attenuated total reflectance (ATR) at room temperature in a range of 4000–400 cm^−1^. UV-Vis absorption spectra were obtained using V-780 spectrophotometer (JASCO, Madrid, Spain) and UV-1800 spectrophotometer (Shimadzu, Kioto, Japan), using quartz cuvettes with a 1 cm path length. Absorption spectra were determined in (4:1) DMSO: deionized H_2_O solutions. Fluorescence spectra were measured using a Varian Cary Eclipse (Agilent, Santa Clara, CA, USA) and quartz cuvettes with a 1 cm path length and 1 mL volume. Contact angles (θ) were measured in air with high purity deionized H_2_O by a 3 μL drop using a contact angle goniometer (THETALITE 100 with the software OneAttension, Espoo, Finland), in order to determine the hydrophobicity of the modified surfaces. Values of the contact angle on at least three samples were measured to give statistical significance. Microwave synthesis was done in a Monowave-400 from Anton Paar (Graz, Austria). Elemental Analysis was determined using Thermo Carlo Erba Flash 2000 at London Metropolitan University. Particle counting was performed by using 10 μL of dispersed microparticles on deionized H_2_O disposed on a Neubauer Chamber from Brand with 0.100 mm Depth and 0.0025 mm^2^ square size. The HPLC system used consisted of a Waters 600 pump and injector, with an Autosampler (717 plus), a 2996 Photo Diode Array and 2475 Multi λ Fluorescence detector. This system was equipped with an *Atlantis* dC18 5 μm pore column (150 × 3.9 mm). The analytical methods used were previously validated. For the analysis of **A**, the mobile phase consisted of a mixture of deionized H_2_O (Channel A), CH_3_CN (Channel B) and CH_3_COONH_4_ (100 mM, pH = 5) (Channel C). The method used was an Isocratic with a proportion of Channel A:Channel B:Channel C (88:2:10%) at flow rate of 1 mL/min and injection volume (10 μL). Samples were monitored and further analyzed at absorption wavelengths of 260–280 nm and 479 nm and emission wavelength of 315 nm.

### 2.3. Synthesis and Characterization of Compounds Based on Bipyridinium Salts

Arbitrary assignment of the proton and carbon atoms of **1·4PF_6_**–**4·4PF_6_** is described in the Supplementary Information (Figure S1).

#### 2.3.1. Synthesis of 1,3-Bis(1′-methyl-4,4′-bipyridiniummethylene) Benzene-Tetrakis (Hexafluorophosphate) (**1·4PF_6_**)

A solution of **5** (1.0 g, 1.7 mmol) in CH_3_NO_2_ (45 mL) was added to a solution of iodomethane (1.4 g, 10.2 mmol) in CH_3_NO_2_ (4 mL). The mixture was heated at 70 °C for 48 h. After cooling down to room temperature, the solvent was evaporated under vacuum. The yellow residue was then dissolved in H_2_O (20 mL), and a saturated aqueous solution of NH_4_PF_6_ (1.7 g, 10.4 mmol, 3 mL) was added. The suspension was kept under strong stirring until no further precipitation was observed. Then, the suspension was filtered off and the solid was washed with H_2_O (30 mL) and dried under vacuum to afford **1**·**4PF_6_** (1.5 g, 87%); mp = 245 °C. ^1^H NMR (400 MHz, (CD_3_)_2_SO, 25 °C): δ 9.43 (d, *J* = 8 Hz; 4H, H-9, 9′, 15, 15′), 9.26 (d, *J* = 8 Hz; 4H, H-14, 14′, 20, 20′), 8.77 (d, *J* = 8 Hz; 4H, H-10, 10′, 16, 16′) 8.71 (d, *J* = 8 Hz; 4H, H-13, 13′, 19, 19′), 7.81 (s; 1H, H-2), 7.55 (m; 3H, H-4, 5, 6), 5.93 (s; 4H, H-7, 8), 4.41 (s; 6H, N–*CH*_3_). ^13^C NMR (400 MHz, (CD_3_)_2_SO, 25 °C): δ 149.9 (C-11, 12, 17, 18), 148.5 (C1, C3), 146.9 (C-14, 14′, 20, 20′), 146.1 (C-9, 9′, 15, 15′), 135.4 (C2), 130.8 (C5), 130.1 (C4, C6), 127.4 (C-13, 13′, 19, 19′), 126.4 (C-10, 10′, 16, 16′), 63.8 (C7, C8), 48.7 (N–*CH*_3_). MALDI-TOF–MS m/z: 881.1 (30%) [M-1PF_6_]^+^, 736.2 (40%) [M-2PF_6_]^+^, 591.22 (100%) [M-3PF_6_]^+^. HMRS (ESI) m/z: [C_30_H_30_F_12_N_4_P_2_]^2+^ calc. 368.09, found 368.08. FT-IR spectrum (KBr, cm^−1^): 2920–2850 ν(C–H) (alkane), 1641 ν(C=N) and 835 δ(C=C).

#### 2.3.2. Synthesis of 1,3-Bis(1′-hexadecyl-4,4′-bipyridiniummethylene) Benzene-Tetrakis (Hexafluorophosphate) (**3·4PF_6_**)

A solution of hexadecylbromide (2.1 g, 7.0 mmol) in DMF (40 mL) was added to a solution of potassium iodide (0.5 g, 2.8 mmol) in DMF (4 mL). The mixture was heated at 80 °C and then, **5** (0.4 g, 0.7 mmol) was added portion-wise. The mixture was stirred for 7 days. After cooling down to room temperature, the orange precipitate was filtered off and washed with CH_3_CN (10 mL) and dried under *vacuum*. The residue was then dissolved in DCM (4 mL) and a saturated aqueous solution of NH_4_PF_6_ (0.5 g, 2.8 mmol, 1 mL) was added until no further precipitation was observed. The suspension was filtered off and the white solid was washed with H_2_O (30 mL), filtered off and dried under vacuum to afford **3**·**4PF_6_** (0.7 g, 85%); mp = 280 °C. ^1^H NMR (400 MHz, (CD_3_)_2_SO, 25 °C): δ 9.41 (d, *J* = 8 Hz; 8H, H-9, 9′, 15, 15′, 14, 14′, 20, 20′), 8.72 (d, *J* = 8 Hz; 8H, H-10, 10′, 16, 16′, 13, 13′, 19, 19′), 7.78 (d, *J* = 8 Hz; 2H, H-4, 6), 7.60 (t, *J* = 8 Hz; 1H, H-5), 7.46 (s; 1H, H-2), 5.97 (s; 8H, –*CH*_2_–), 2.09 (s; 4H, H-7, 8), 1.24 (s; 52H, –*CH*_2_–), 0.85 (s; 6H, –*CH*_3_). ^13^C NMR (400 MHz, (CD_3_)_2_SO, 25 °C): δ 149.7 (C-11, 12, 17, 18), 148.7 (C1, C3), 146.4 (C-14, 14′, 20, 20′), 146.2 (C-9, 9′, 15, 15′), 135.2 (C2), 130.1 (C5), 127.5 (C4, C6), 127.2 (C-13, 13′, 19, 19′), 127.0 (C-10, 10′, 16, 16′), 63.4 (C7), 61.4 (C8), 31.7–22.9 (*(CH*_2_*)*_14_), 14.4 (*CH*_3_). MALDI-TOF–MS m/z: 1156.6 (3%) [M-2PF_6_]^+^, 1011.6 (100%) [M-3PF_6_]^+^, 866.6 (30%) [M-4PF_6_]^+^. HMRS (ESI) m/z: [C_60_H_90_F_12_N_4_P_2_]^2+^ calc. 578.33, found 578.32. FT-IR spectrum (KBr, cm^−1^): 2918–2851 ν(C–H) (alkane), 1642 ν(C=N) and 837 δ(C=C).

#### 2.3.3. Synthesis of 1,3-Bis(1′-methyl-4,4′-bipyridiniummethylene)-5-propargyloxybenzene-tetrakis (Hexafluorophosphate) (**4·4PF_6_**)

In a microwave tube (10 mL), *N*-methyl-4,4′-bipyridinium iodide (0.2 g, 0.6 mmol), 1,3-dibromomethyl-5-propargyloxybenzene (0.09 g, 0.3 mmol) and CH_3_CN (5 mL) were added. The mixture was irradiated in a microwave oven at 130 °C for 10 min. After that, a brownish precipitated was obtained and the final suspension was centrifuged with CH_3_CN (3 × 5 mL). The brown residue was then dissolved in H_2_O (15 mL), and NH_4_PF_6_ (260 mg, 1.6 mmol) was added. The suspension was kept under stirring until no further precipitation was observed and filtered off. The pale-yellow solid was washed with H_2_O (20 mL) and with Et_2_O (2 mL), filtered off and dried under vacuum to afford **4**·**4PF_6_** (0.3 g, 81%); mp = 250 °C. ^1^H NMR (250 MHz, DMSO, 25 °C): δ 9.44 (d, *J* = 7 Hz; 4H, H-9, 9′, 15, 15′), 9.28 (d, *J* = 7 Hz; 4H, H-14, 14′, 20, 20′), 8.78 (d, *J* = 7 Hz; 4H, H-10, 10′, 16, 16′), 8.72 (d, *J* = 7 Hz; 4H, H-13, 13′, 19, 19′), 7.44 (s; 1H, H2), 7.26 (s; 2H, H-4, 6), 5.91 (s; 4H, H-7, 8), 4.83 (d, *J* = 2 Hz; 2H, O–*CH*_2_–), 4.44 (s; 6H, N–*CH*_3_), 3.59 (t, *J* = 2 Hz; 1H, CH_2_–*C*≡*CH*). ^13^C NMR (250 MHz, DMSO, 25 °C): δ 158.1 (C5), 149.3 (C11, C17), 148.1 (C12, C18), 146.7 (C-14, 14′, 20, 20′), 145.9 (C-9, 9′, 15, 15′), 136.2 (C1, C3), 126.9 (C-10, 10′, 16, 16′), 126.2 (C-13, 13′, 19, 19′), 122.3 (C2), 116.1 (C4, C6), 78.9 (C13), 78.5 (–*C*≡*CH*), 63,0 (C7, C8), 55.8 (O–*CH*_2_–), 48.1 (N–*CH*_3_). HRMS (ESI) m/z: [C_33_H_32_N_4_O]^4+^ calc. 125.06, found 125.06. FT-IR (ATR) (cm^−1^): 3253 ν(C–H) (alkyne), 3031–2982 ν(C–H) (alkane), 2127 ν(C≡C), 1634 ν(C=N), 1556 ν(C=C) and 819 δ(C=C). 

### 2.4. Formation of π-Acceptor/π-Donor Complexes between **1·4PF_6_**–**4·4PF_6_** and Neurotransmitters: **D**, **S**, **A** and **NA** in Solution

For **1**·**4PF_6_**–**3**·**4PF_6_**_,_ a solution of either **D** (0.2 mg, 1.1 μmol), **S** (0.2 mg, 1 μmol), **A** (0.2 mg, 0.9 μmol) or **NA** (0.2 mg, 0.9 μmol) in deionized H_2_O (0.5 mL) was added to a solution of either **1**·**4PF_6_** (4.2 mg, 4 μmol), **2**·**4PF_6_** (4.8 mg, 4 μmol), **3**·**4PF_6_** (5.6 mg, 3.9 μmol) in DMSO (2 mL). The mixtures were stirred at 25 °C for 24 h under dark. 

For **4**·**4PF_6_**, a solution of either **D** (0.9 mg, 5 μmol), **S** (1.1 mg, 5 μmol), **A** (1.1 mg, 5 μmol) or **NA** (1.0 mg, 5 μmol) in deionized H_2_O (0.5 mL) was added to a solution of **4**·**4PF_6_** (5.4 mg, 5 μmol) in DMSO (2 mL), being the final concentration for each component 2 mM. The mixtures were stirred at 25 °C for 24 h under dark.

### 2.5. Functionalization of Polysilicon Surfaces (Poly-Surfs) with Bipyridinium Salts **1·4PF_6_**–**4·4PF_6_**, and Incorporation of **D**, **S**, **A** and **NA**

#### 2.5.1. Cleaning and Activation Surface Protocol

The polysilicon surfaces were immersed in a freshly prepared piranha solution, H_2_SO_4_: H_2_O_2_ at a volume ratio of 7:3 (1.4, 0.6 mL, respectively) during 1.5 h at room temperature. Then, the substrates were rinsed with deionized H_2_O (2 × 2 mL), dried with N_2_ flow and immediately immersed in an alkaline mixture of NH_4_OH: H_2_O_2_: H_2_O, at a volume ratio 1:1:5 (0.3, 0.3, 1.4 mL, respectively) for 30 min. The substrates were rinsed with deionized H_2_O (2 × 2 mL) and dried with N_2_ flow.

#### 2.5.2. Non-Covalent Immobilization of **1·4PF_6_**–**3·4PF_6_** and Incorporation of **D**, **S**, **A** and **NA**

The hydroxylated surfaces were immediately immersed in a solution of either **1**·**4PF_6_** (2.1 mg, 1 mM), **2**·**4PF_6_** (2.4 mg, 1 mM) or **3**·**4PF_6_** (2.9 mg, 1 mM) in DMSO (2 mL) and were stirred at 90 rpm for 6 h. After the deposition time, the substrates were rinsed with DMSO (5 × 3 mL) and deionized H_2_O (5 × 3 mL) and dried with N_2_ flow. The functionalized substrates with either **1**·**4PF_6_**, **2**·**4PF_6_**, **3**·**4PF_6_** were immersed in a solution of either **D** (0.8 mg, 2 mM), **S** (0.9 mg, 2 mM), **A** (0.9 mg, 2 mM) or **NA** (0.8 mg, 2 mM) in deionized H_2_O (2 mL) and were stirred at 90 rpm for 24 h. After this time, the substrates were rinsed with deionized H_2_O (5 × 3 mL) and dried with N_2_ flow.

#### 2.5.3. Covalent Immobilization of **4·4PF_6_** and Incorporation of **D**, **S**, A and **NA**

The hydroxylated surfaces were immersed in a solution of 11-azidoundecyltrimethoxysilane (20 μL, 30.6 mM) in toluene (2 mL) and stirred at 90 rpm overnight, rinsed with toluene (2 × 2 mL) and EtOH (2 × 2 mL) and dried with N_2_ flow. Then, the azido-functionalized surfaces were immersed in a mixture of **4**·**4PF_6_** (4.3 mg, 1 mM), CuSO_4_ (64 μg, 0.1 mM) and sodium ascorbate (0.8 mg, 1 mM) in EtOH: H_2_O: DMSO at volume ratio (3 mL, 0.6 mL, 0.4 mL) and stirred at 90 rpm overnight at 40 °C. After this time, the substrates were rinsed with EtOH (2 × 4 mL) and deionized H_2_O (2 × 4 mL) and dried with N_2_ flow. The functionalized substrates with **4**·**4PF_6_** were immersed in a solution of either **D** (1.9 mg, 2 mM), **S** (2.1 mg, 2 mM), **A** (2.2 mg, 2 mM) or **NA** (2.1 mg, 2 mM) in deionized H_2_O (5 mL) and were stirred at 90 rpm for 24 h. After this time, the substrates were rinsed with deionized H_2_O (2 × 5 mL) and dried with N_2_ flow.

#### 2.5.4. Control Surfaces with **D**, **S**, **A** and **NA**

The hydroxylated polysilicon surfaces used as control experiments were separately immersed in a solution of either neurotransmitter (2 mM) in deionized H_2_O (2 mL) for 24 h. After this time, the substrates were rinsed with deionized H_2_O (2 × 5 mL) and dried with N_2_ flow.

### 2.6. Functionalization of Polysilicon Microparticles (Poly-Si μPs) with **1·4PF_6_** and **4·4PF_6_** and Incorporation of **A**

#### 2.6.1. Cleaning and Activation Protocol of Poly-Si μPs 

A total of 100 μL of a freshly prepared piranha solution (H_2_SO_4_:H_2_O_2_) at volume ratio of 7:3 (0.7 mL, 0.3 mL, respectively) was added to different microtubes containing 1.5 × 10^6^ polysilicon microparticles. The suspension was incubated for 1.5 h at room temperature while stirred on a vortex mixer at 400 rpm and then centrifuged (13,500 rpm, 15 min), forming a pellet of microparticles, which were resuspended and washed with deionized H_2_O (2 × 100 μL). The suspension was centrifuged (13,500 rpm, 10 min) forming a pellet of microparticles and the supernatant was removed. Then, 100 μL of a freshly prepared alkaline mixture of NH_4_OH: H_2_O_2_: H_2_O, at a volume ratio 1:1:5 (0.1 mL, 0.1 mL, 0.7 mL, respectively) was added to the pellet. After resuspending the microparticles, they were stirred on a vortex mixer at 400 rpm for 30 minutes. After this time, the suspension was centrifuged (13,500 rpm, 15 min) and washed with deionized H_2_O (2 × 100 μL) and EtOH (100 μL), centrifuging (13,500 rpm, 10 min) after each washing step and removing the supernatant.

#### 2.6.2. Non-Covalent Immobilization of **1·4PF_6_** on Poly-Si μPs 

A total of 100 μL of a solution of **1**·**4PF_6_** (2.1 mg, 1 mM) in DMSO (2 mL) was added to the pellet of the hydroxylated microparticles. The microtubes containing the microparticles were stirred at 400 rpm overnight. After the deposition time, microparticles were sedimented by centrifugation (13,500 rpm, 10 min) and washed with DMSO (5 × 100 μL).

#### 2.6.3. Covalent Immobilization of **4·4PF_6_** on Poly-Si μPs 

The hydroxylated microparticles were immersed in 100 μL of a solution of 11-azidoundecyltrimethoxysilane (10 μL, 15.3 mM) in toluene (1 mL) and stirred on a vortex mixer at 400 rpm overnight. The suspension was then centrifuged (13,500 rpm, 10 min), washed with toluene (2 × 100 μL) and EtOH (100 μL), centrifuging (13,500 rpm, 10 min) after each washing step and removing the supernatant. After that, to the azido-functionalized microparticles, 200 μL of a mixture of **4**·**4PF_6_** (1.08 mg, 1 mM), CuSO_4_ (0.032 mg, 0.1 mM) and sodium ascorbate (0.2 mg, 1 mM) in EtOH: H_2_O:DMSO (0.5, 0.3, 0.2 mL) were added. The microtubes containing the microparticles were stirred on a vortex mixer at 400 rpm overnight at 40 °C. Then, the microparticles were sedimented by centrifugation (13,500 rpm, 10 min) and the supernatant was removed. The microparticles were washed with EtOH (2 × 200 μL) and deionized H_2_O (2 × 200 μL), centrifuging (13,500 rpm, 10 min) after each washing step and removing the supernatant.

#### 2.6.4. Incorporation of A in **1·4PF_6_** and **4·4PF_6_** Functionalized Poly-Si μPs 

A total of 200 μL of an aqueous solution of **A** (2.64 mg, 2mM) in deionized H_2_O (6 mL) was added to the suspension of polysilicon microparticles functionalized with **1**·**4PF_6_** or **4**·**4PF_6_**. All microtubes (four microtubes for each **1**·**4PF_6_** and **4**·**4PF_6_**, respectively) were stirred on a vortex mixer at 400 rpm for 24 h at room temperature. After this time, microparticles were sedimented by centrifugation (13,500 rpm, 10 min) and washed with deionized H_2_O (3 × 200 μL), centrifuging (13,500 rpm, 10 min) after each washing step and removing the supernatant.

### 2.7. Quantification of A Incorporated in Poly-Si μPs by HPLC Determination

After the incubation of the functionalized microparticles (**1**·**4PF_6_** or **4**·**4PF_6_**) with **A**, the suspensions of the four microtubes were centrifuged (13,500 rpm, 10 min) and the supernatant of each microtube was kept. A total of 100 μL of each supernatant solution was diluted up to 1 mL with deionized H_2_O (900 μL), considered as the final solution (**A_fin_**). Moreover, 100 μL of the 2 mM solution of **A** in deionized H_2_O were diluted up to 1 mL, with deionized H_2_O (900 μL) being the initial solution (**A_in_**). These **A_in_** and **A_fin_** solutions were analyzed by HPLC coupled to a fluorescence detector (λ_exc_ = 280 nm, λ_em_ = 315 nm). The retention time of **A** was 2.5 min and the area under the curve (AUC) of the retention peaks was measured. The amount of **A** incorporated was estimated using a calibration curve of **A** in a range of 0.18–7.21 × 10^−4^ mM in deionized H_2_O.

In order to calculate the final amount of microparticles, they were suspended in 100 μL of deionized H_2_O. A total of 10 μL of each suspension was deposited in a Neubauer chamber and images by optical microscope were collected.

### 2.8. Quantification of A Released from Poly-Si μPs by HPLC Determination

The microparticles functionalized with either **1**·**4PF_6_**:**A** or **4**·**4PF_6_**:**A** of the four microtubes used were collected in a single microtube and centrifuged (13,000 rpm, 10 min). A total of 200 μL of a solution of ascorbic acid (4 mM) in deionized H_2_O (200 μL) was added to the precipitated microparticles with either **1**·**4PF_6_**:**A** or **4**·**4PF_6_**:**A**. The microparticles were then suspended by sonication (1 min) and the suspension was incubated for 20 min at room temperature without stirring. Afterwards, microparticles were sedimented by centrifugation (13,000 rpm, 10 min), and the supernatant was collected (release solution—T1). The release process with ascorbic acid was repeated and a second release solution (release solution—T2) was collected. The release solutions (T1 and T2) were analyzed by HPLC coupled to a fluorescence detector (λ_exc_ = 280 nm, λ_em_ = 315 nm). The retention time of **A** was 2.5 min and the area under the curve (AUC) of the retention peaks was measured. The amount of **A** released was estimated using a calibration curve of **A** in a range of 0.18–7.21 × 10^−4^ mM in deionized H_2_O.

## 3. Results and Discussion

### 3.1. Synthesis of **1·4PF_6_**–**4·4PF_6_**

In this work, four π-deficient bipyridinium salts (**1**·**4PF_6_**–**4**·**4PF_6_**) have been synthesized and characterized. These π-deficient salts were used as hosts in the non-covalent and covalent functionalization of polysilicon surfaces and microparticles for the subsequent encapsulation and release of π-excessive neurotransmitters. These π-deficient systems are composed of two bipyridinium units linked through a 1,3-bis(methylene)benzene spacer. The **1**·**4PF_6_**–**3**·**4PF_6_** salts can be chemisorbed directly on polycrystalline silicon surfaces, whereas the incorporation of a propargyloxy moiety in **4**·**4PF_6_** allows its covalent immobilization by copper(I)-catalyzed azide-alkyne cycloaddition (CuAAC).

**Scheme 1:**
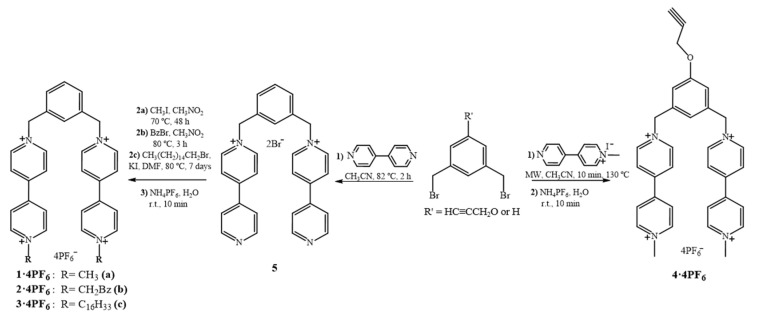
Synthesis of **5** and bipyridinium salts **1·4PF_6_**, **2·4PF_6_**, **3·4PF_6_** and **4·4PF_6_**.

The synthesis of **1·4PF_6_**–**4·4PF_6_** salts is presented in Scheme 1. Compounds **5** and **2·4PF_6_** were prepared following protocols previously described in the literature [25,43] (see Supporting Information). On the other hand, the bipyridinium salts **1·4PF_6_** and **3·4PF_6_** were obtained by reaction of **5** with the corresponding alkyl halide, iodomethane or hexadecylbromide, obtaining 87% and 85% of yield, respectively, after anion exchange with NH_4_PF_6_. For **4·4PF_6_**, microwave (MW) radiation was used to perform the reaction between the *N*-methyl-4,4′-bipyridinium iodide and 1,3-dibromomethyl-5-propargyloxy benzene to obtain the corresponding hexafluorophosphate salt with 81% of yield, after anion exchange with NH_4_PF_6_ [44].

The bipyridinium salts **1**·**4PF_6_**–**4**·**4PF_6_** were characterized by ^1^H and ^13^C NMR spectroscopy, MALDI-TOF or HR-ESI mass spectrometry and Infrared (FT-IR) spectroscopy (see Supporting Information, Figures S1–S21).

### 3.2. Characterization of Neurotransmitters Complexation with **1·4PF_6_**–**4·4PF_6_** in Solution Using Ultraviolet-Visible Absorption and Fluorescence Spectroscopies

**Figure 2:**
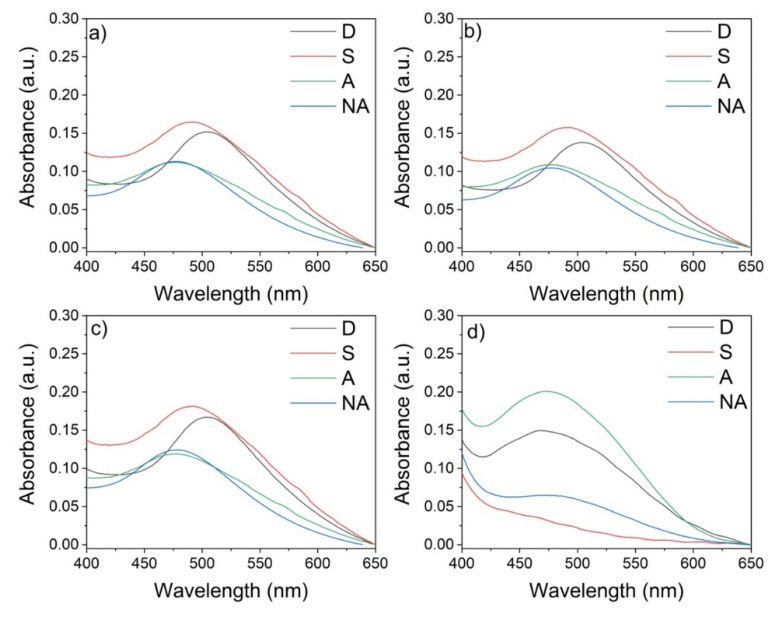
UV-Vis absorption spectra of a mixture of **D**, **S**, **A** or **NA** with: (**a**) **1·4PF_6_**, (**b**) **2·4PF_6_**, (**c**) **3·4PF_6_** and (**d**) **4·4PF_6_** in 2.5 mL of DMSO:H_2_O (4:1). The spectra show the corresponding charge-transfer absorption band.

Four π-donor neurotransmitters (**D**, **S**, **A** and **NA**) were selected in this work, to be encapsulated on Poly-Surfs and Poly-Si μPs. This π-donor nature favors the interaction with the π-acceptor bipyridinium salts used as hosts in the non-covalent and covalent functionalization of the polysilicon substrates. The complexation between the neurotransmitters and the π-acceptor hosts (**1**·**4PF_6_**–**4**·**4PF_6_**) was initially followed by ultraviolet-visible (UV-Vis) absorption spectroscopy in solution. Therefore, the appearance of the corresponding visible charge-transfer (CT) band upon mixing either of **1**·**4PF_6_**–**4**·**4PF_6_** in DMSO with either **D**, **S**, **A** or **NA** in deionized H_2_O, indicated the formation of the π-acceptor/π-donor complexes. The use of the mixture DMSO: H_2_O (4:1) responds to the insolubility of the π-acceptor bipyridinium salts in aqueous solutions. To the naked eye, the complex formation was also evidenced by a solution color change from pale yellow to pink. Figure 2 shows the UV-Vis absorption spectra for the association of the tetracationic hosts **1**·**4PF_6_**, **2**·**4PF_6_**, **3**·**4PF_6_** or **4**·**4PF_6_** with **D**, **S**, **A** and **NA** (Figure 2a–d, respectively). For all cases, the characteristic CT bands were observed in a range between 400–600 nm, as it has been reported for similar structures [20]. In this UV-Vis region none of the compounds have an absorbance band (see Supporting Information Figure S22). Furthermore, the results show that **1**·**4PF_6_**–**3**·**4PF_6_** present higher incorporation of **S**, whereas **4**·**4PF_6_** presents a higher interaction with **A**.

Fluorescence spectroscopy was also used to monitor the incorporation of the neurotransmitters in solution. Due to the similar response observed for **1**·**4PF_6_**–**3**·**4PF_6_** with the neurotransmitters by UV-Vis studies, the fluorescence emission studies were performed using only **1**·**4PF_6_** and **4**·**4PF_6_**, which have similar structures, and will allow the comparison of the non-covalent and covalent functionalization of the Poly-Surfs. The fluorescence spectra of the neurotransmitters and **1**·**4PF_6_** and **4**·**4PF_6_**, respectively, in DMSO: H_2_O (4:1) solution depicted that the bipyridinium salts are not fluorescent (see Supporting Information Figure S23a), whereas all catecholamine-based neurotransmitters (**D**, **A** and **NA**) presented an emission maximum peak at 315 nm, the indolamine-based neurotransmitter (**S**) presented an emission maximum peak at 338 nm (see Supporting Information Figure S23a). Once the neurotransmitters were mixed with either **1**·**4PF_6_** and **4**·**4PF_6_**, a decrease in their fluorescence intensity was observed, suggesting that the formation of the complex induced fluorescence emission quenching (see Supporting Information Figure S23b,c). All fluorescence spectra were collected at the maximum excitation wavelengths (see Supporting Information, Figure S22).

### 3.3. Functionalization and Characterization of Polysilicon Surfaces and Microparticles with **1·4PF_6_**–**4·4PF_6_** and Neurotransmitter Incorporation

**Figure 3:**
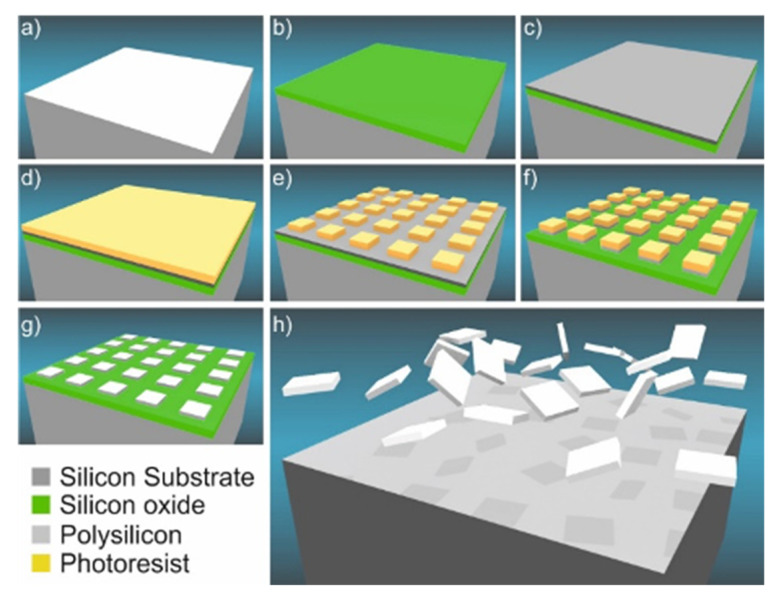
Fabrication of the polysilicon substrates. (**a**) Silicon wafer starting substrate. (**b**) Thermal growing of a 1 µm-thick silicon oxide (sacrificial layer). (**c**) Deposition of a 500 nm-thick CVD polysilicon layer (structural layer). (**d**) 1.2 µm-thick photoresist layer deposition. (**e**) Photolithographic step to define lateral dimensions. (**f**) Vertical dry etching of the polysilicon layer. (**g**) Removing of the photoresist layer to obtain the Poly-Surfs. (**h**) Etching of the sacrificial layer in HF vapors and release of Poly-Si μPs.

Poly-Surfs and Poly-Si μPs, which are robust and biocompatible materials, were selected to be functionalized with **1·4PF_6_**–**3·4PF_6_** and **4·4PF_6_** [14,15]. Hence, functionalized polysilicon substrates were explored as carriers for the encapsulation of neurotransmitters (**D**, **S**, **A** or **NA**). The fabrication process of both polysilicon substrates was performed following the protocols previously reported [12,46]. Briefly, a four-inch p-type silicon wafer is used as a starting substrate (Figure 3a). The fabrication process is based in the combination of a 500 nm-thick polysilicon device layer deposited by chemical vapor deposition (CVD) on a 1 µm thermal grown silicon oxide layer that acts as a sacrificial layer (Figure 3b,c). Then, 1.2 µm of photoresist was spun on the polysilicon layer (Figure 3d), to subsequently define the lateral dimensions of the device by a photolithographic step (Figure 3e) followed by a vertical polysilicon dry etching (Figure 3f). Afterwards, the photoresist was removed by plasma etching (Figure 3g). An array of 3 × 3 µm separated 3 µm was obtained (Poly-Surfs). To obtain the Poly-Si μPs, they were released from the array by the etching of the silicon oxide sacrificial layer in vapors of hydrofluoric acid (HF) (Figure 3h). Finally, the released microparticles were suspended in ethanol by using an ultrasonic bath and collected by 5 µm filter rating. Three medium changes are made to the suspended particles by centrifugation to ensure removal of possible traces of HF. 

**Figure 4:**
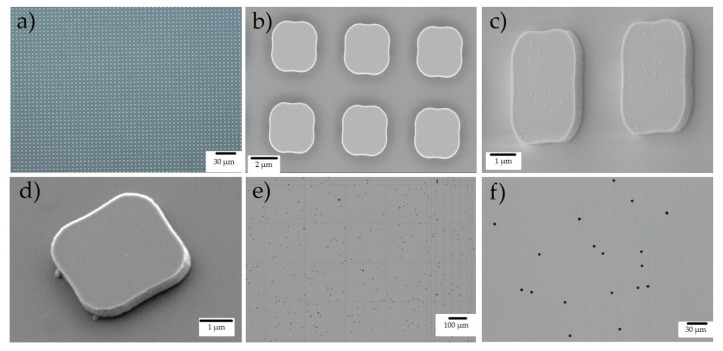
Images of Poly-Surfs with patterned Poly-Si μPs with lateral dimensions 3 × 3 μm^2^ and 0.5 μm thickness on a thermal silicon oxide layer: (**a**) Optical microscope image at scale bar of 30 μm); (**b**,**c**) Scanning electron microscope (SEM) images at scale bars of 2 and 1 μm. Images of etched and suspended Poly-Si μPs: (**d**) SEM image of a single Poly-Si μP; (**e**,**f**) Optical microscope images at scale bar of 100 and 30 μm, respectively.

Therefore, the Poly-Surfs used have an area of approximately 1 cm^2^ and present a pattern of polysilicon microparticles with lateral dimensions 3 × 3 μm^2^ and 0.5 μm thickness on the thermal silicon oxide layer (Figure 4a–c). Moreover, the bipyridinium salts **1·4PF_6_** and **4·4PF_6_** were also immobilized on suspended microparticles of 3 × 3 μm^2^ and 0.5 μm thickness (Figure 4d–f). Particle size distribution of the Poly-Surfs and Poly-Si μPs suspended in solution were performed, analyzing scanning electron microscope (SEM) and optical microscope images, respectively. The statistics particle size histogram showed an average length of 3.24 ± 0.07 μm for the Poly-Surfs and 3.34 ± 0.11 μm for the Poly-Si μPs (Figure S24). The deviation of each measurement confirmed the high reproducibility of the microparticles achieved using the microfabrication techniques. These dimensions were chosen due to the demonstrated internalization of microparticles with this size into cells and their low toxicity [15]. 

The functionalization was always first attempted using the Poly-Surfs and the synthetic methodology was then adapted to the Poly-Si μPs’ functionalization. Since single-microparticle surface characterization based on common laboratory techniques together with the shape of the Poly-Si μPs is still challenging, the use of the polysilicon surfaces has facilitated the final microparticle surface functionalization and characterization. 

**Scheme 2:**
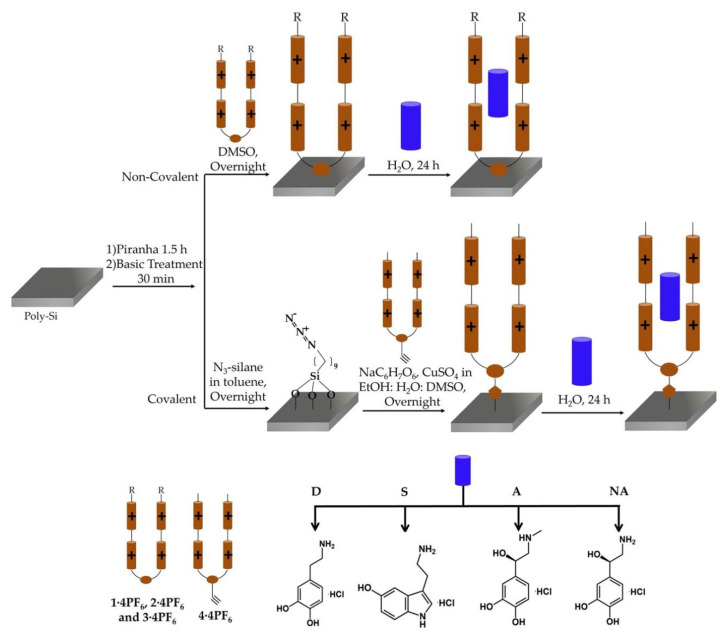
Functionalization of polysilicon surfaces (Poly-Surfs) and microparticles (Poly-Si μPs) with bipyridinium salts **1**·**4PF_6_**–**4**·**4PF_6_** and the subsequent incorporation of **D**, **S**, **A** or **NA**. R = CH_3_ (**1·4PF_6_**); CH_2_Bz (**2·4PF_6_**); C_16_H_33_ (**3·4PF_6_**).

Scheme 2 presents the two different approaches used for the immobilization of the host molecules on the Poly-Surfs, the non-covalent with **1**·**4PF_6_**–**3**·**4PF_6_** and the covalent with **4**·**4PF_6_**. On both strategies, the first step consisted on cleaning and activating the polycrystalline silicon substrates using piranha solution and basic piranha solution. Then, the Poly-Surfs were functionalized by immersing the substrates into a DMSO solution of either **1**·**4PF_6_**–**3**·**4PF_6_**. For the covalent immobilization of **4**·**4PF_6_**, first an azide-terminated monolayer was prepared using 11-azidoundecyltrimethoxysilane (N_3_-silane) followed by the azide-alkyne cycloaddition between the azido-functionalized surface and the alkyne moiety of **4**·**4PF_6_**. Finally, functionalized substrates with the corresponding bipyridinium salts (**1**·**4PF_6_**–**4**·**4PF_6_**) were immersed in an aqueous solution of either **D**, **S**, **A** or **NA** to be encapsulated. Additionally, control experiments were made by the immersion of activated Poly-Surfs in a solution of each neurotransmitter (**D**, **S**, **A** or **NA**).

The functionalization of the Poly-Surfs and the incorporation of the neurotransmitters were characterized by contact angle (θ) measurements, and the values obtained in all functionalization steps are shown in Table 1. The initial polysilicon surfaces exhibit a water contact angle of 44° ± 3. After cleaning and activation steps, a decrease in the contact angle values was observed, obtaining values of 16° ± 2 and 10° ± 1, respectively, and confirming the high hydrophobicity of the surfaces. Functionalized polysilicon surfaces with **1**·**4PF_6_**–**3**·**4PF_6_** showed an increase in the contact angle values between θ = 57°–89° ± 1, due to the hydrophobic character of the bipyridinium salts (see Table 1). Moreover, the surface functionalized with **3**·**4PF_6_** presented a higher contact angle, due to the longer alkyl chain of 16 carbons, than the rest of the immobilized bipyridinium salts. These results suggest that the polysilicon surfaces were successfully functionalized, and also indicates that the part of the molecule which is facing outwards to the polysilicon surface is the aliphatic moiety, while the polar head is adsorbed onto the surface. 

On the other hand, for the covalent approach, the azido-functionalization presented an hydrophobic contact angle with a value of 78° ± 1, typical for terminal azide-monolayers [47]. After the 1,3-dipolar cycloaddition reaction, the value decreased to 55° ± 4, which is comparable to the value obtained for **1**·**4PF_6_**, due to the similarities in their structure, suggesting the formation of the **4**·**4PF_6_** layer [48]. After the incorporation of either of the four different water-soluble neurotransmitters (**D**, **S**, **A** or **NA**) on the functionalized polysilicon surfaces, the contact angle values decrease noticeably, as shown in Table 1, indicating the presence of the water-soluble π-electron rich neurotransmitters. This result denotes that the π-donor compounds were incorporated on the polysilicon surfaces through an aromatic π-acceptor/π-donor complex. For the control experiments, an increase in the contact angle values was observed compared to the activation step, suggesting the adsorption of the neurotransmitters onto the activated surfaces. However, the contact angles values were lower than the ones obtained when the bipyridinium salts were immobilized, indicating the successful complex formation [49].

Polysilicon microparticles (3 × 3 μm^2^ and 0.5 μm thickness) were then functionalized in suspension with either **1**·**4PF_6_** or **4**·**4PF_6_**, both having the same peripheral ending group, in order two compare both strategies of functionalization (non-covalent and covalent). The functionalization protocols followed for these substrates were similar to the ones optimized for the functionalization of the Poly-Surfs (Scheme 2). The π-donor neurotransmitter **A** was then selected for its encapsulation on the Poly-Si μPs functionalized with **1**·**4PF_6_** or **4**·**4PF_6_**. 

High-performance liquid chromatography coupled to a fluorescence detector (HPLC-FLD) was used along the experiments with microparticles for the quantification of **A** that is incorporated in them, using excitation and emission wavelengths of 280 and 315 nm, respectively. Therefore, to determine the amount of **A** that can be encapsulated in the microparticles functionalized with either **1·4PF_6_** or **4·4PF_6_**, a 2 mM solution of **A** was first prepared (**A_in_** or initial solution). Then, the Poly-Si μPs functionalized with **1·4PF_6_** or **4·4PF_6_** were immersed in the **A_in_** solution for 24 h. After this time, microparticles were sedimented by centrifugation and the supernatant was collected (**A _fin_** or final solution). Before **A _in_** and **A _fin_** solutions were injected in the HPLC, a dilution (1:10) was necessary in order to avoid the saturation of the fluorescence detector. Hence, the concentrations of the initial and final solutions were obtained by means of a calibration curve of **A** in the range of 0.18–7.21 × 10^−4^ mM in deionized H_2_O (see Supporting Information, Figures S25 and S26 and Table S1), and the amount of **A** incorporated into the functionalized microparticles was determined by difference. Moreover, in order to estimate the average **A** encapsulated per microparticle, the number of microparticles corresponding to each suspension was also determined by optical microscopy, using a Neubauer chamber (see experimental details, Figure 4e and Equation S1). 

The results show that the Poly-Si μPs functionalized with **4**·**4PF_6_** can incorporate almost two times more **A** per microparticle (7.8 × 10^−8^ μmol/particle) than those functionalized with **1**·**4PF_6_** (4.3 × 10^−8^ μmol/particle) (see Supporting Information Table S2). This trend was also observed in the UV-Vis solution experiments, showing that the structural difference between both molecules may have an influence on the host–guest complex formation. 

### 3.4. Release Studies from A Encapsulated in Microparticles

The release of **A** from the functionalized Poly-Si μPs, with **1**·**4PF_6_** or **4**·**4PF_6_**, was carried out using ascorbic acid (Vitamin C) as the release trigger. Ascorbic acid is a natural compound present in cells, and more importantly, capable of reducing bipyridinium salts, and hence, it may disassemble the supramolecular complex formed by **4**·**4PF_6_** and **A**, releasing the encapsulated material [50]. To this end, the dissociation of the **4**·**4PF_6_**:**A** complex was first studied by UV-Vis absorption spectroscopy in solution (see Supporting Information Figure S27). Upon addition of ascorbic acid in a ratio 2:1 (ascorbic: **4**·**4PF_6_**), the color of the solution changed from pink to blue, suggesting the reduction in the viologen moieties of **4**·**4PF_6_** to their radical cation. In this regard, the UV-Vis spectrum shows the disappearance of the charge-transfer band and the appearance of the bands corresponding to the bipyridinium radical cation at 400 and 600 nm, suggesting the disassembly of the complex in presence of the triggering agent (see Supporting Information Figure S27) [51]. Moreover, the broad band from 250 to 350 nm could be attributed to **A** released (279 nm), the presence of dehydroascorbic acid (300 nm), which is the result of the oxidation of the ascorbic acid, and the excess of the triggering agent used (265 nm) [52]. 

HPLC coupled to a UV-Vis absorption spectroscopy detector (HPLC-UV-Vis) was also used to study the encapsulation of **A** through the formation of π-acceptor/π-donor complexes (**1**·**4PF_6_**:**A** or **4**·**4PF_6_**:**A)**, as well as their disassembly under chromatographic conditions. For that, 2 mM solutions of either **A**, **1**·**4PF_6_**, **4**·**4PF_6_**, **1**·**4PF_6_**:**A** and **4**·**4PF_6_**:**A** (2 mM: each) were analyzed separately by monitoring the absorption at 479 nm, corresponding to the wavelength of the charge-transfer band of the complex. Chromatograms of either **A, 1**·**4PF_6_** or **4·4PF_6_** show no peaks (besides the solvent front around 1.5–2 min) (see Supporting Information Figure S28a,b,e). However, when analyzing the chromatograms of the complexes (**1**·**4PF_6_**:**A** and **4**·**4PF_6_**:**A**) (Figure S28c and Figure S28f, respectively) new peaks can be observed at retention times of 7.25 and 7.5 min, respectively. In addition, when extracting the corresponding absorption spectra of the samples at these retention times, the absorption spectra clearly show the charge-transfer band (see Supporting Information, Figure S29c,g). These results confirm the successful formation of the complex and its stability under the chromatographic conditions, while the longer retention times (>7 min) as compared to those of the compounds, show the hydrophobic nature of the π-acceptor/π-donor complexes as compared to the compounds separately. Furthermore, once the trigger agent was added to the complexes **1**·**4PF_6_**:**A** and **4**·**4PF_6_**:**A**, the signal corresponding to the complexes at retention time between 7 and 8 min disappeared, confirming the disassembly of the complex upon the reduction in the bipyridinium moiety (see Supporting Information Figure S28d,g). Likewise, the absorption spectra extracted from those chromatograms at the same retention times do not show the charge-transfer absorption band (between 400–600 nm) (see Supporting Information, Figure S29d,h).

To assess if **A** could be released from the Poly-Si μPs functionalized with the complexes **1**·**4PF_6_**:**A** or **4**·**4PF_6_**:**A**, upon an external stimulus, they were incubated in an aqueous solution of ascorbic acid. In order to calculate the amount of **A** released from the functionalized Poly-Si μPs, they were centrifuged and the supernatant was kept (release solution- T1). Moreover, to ensure the maximum amount of **A** released, a second cycle of incubation with ascorbic acid was carried out, obtaining a second release solution (T2). The amount of **A** in the release solutions (T1 and T2) was then analyzed by HPLC coupled to a fluorescence detector set (λ_exc_ = 280 nm and λ_em_ = 315 nm). Since **A** was the only fluorescent compound in the mixture, the areas under the curve (AUC) of the fluorescent peaks at retention time of 2.5 min were measured and the amount of released **A** was obtained by using the calibration curve (see Supporting Information: Figure S25, Figure S30 and Table S3). Particles functionalized with **1**·**4PF_6_** can release **A** in the presence of ascorbic acid up to 5.0 × 10^−9^ μmol/particle, while those functionalized with **4**·**4PF_6_** can release up to 4.7 × 10^−9^ μmol/particle (see Supporting Information Table S4). However, in global a, higher release of **A** was observed for microparticles functionalized with **1**·**4PF_6_** (9% of **A** encapsulated) than those functionalized with **4**·**4PF_6_** (5% of **A** encapsulated), indicating that, with the covalent approach, a higher amount of the incorporation and lower release of **A** can be assessed (see Supporting Information Table S4).

Finally, in order to ensure that the presence of the ascorbic acid did not affect the physicochemical properties of **A** in the mixture during the release experiments, a stability study was performed. For this purpose, a mixture of **A** and ascorbic acid (1:2 molar ratio) was analyzed every 10 min, for a total of 55 min (the overall time involved in the release experiment) by HPLC-FLD. For all the experiments, the area of the peaks corresponding to **A,** at retention time of 2.5 min, was measured and the concentration of **A** was estimated using the previous calibration curve (see Supporting Information Figure S25). This experiment showed that the concentration of **A** was maintained for at least 55 min, corroborating the stability of **A** during the release experiments (see Supporting Information Figure S31).

### 3.5. Cytotoxicity and Genotoxicity Assay of **1·4PF_6_**

The non-covalent attachment of **1**·**4PF_6_** to the Poly-Si μPs could imply some possible desorption of the compound along time and therefore its toxicity was necessary studied using three different cell lines (3T3/NIH, HepG2 and Caco-2). The cells were exposed to a range of concentrations of compound **1**·**4PF_6_** for 24 h. After exposure, the viability of the cell culture was performed using the MTT Assay, and the percentage of viable cells is shown in the Supporting Information (Tables S5–S7). Moreover, IC_50_ values were calculated for the three different cell lines and are shown in the Supporting Information (Table S8). As can be seen, the IC_50_ values (above 122 μM) found for **1**·**4PF_6_** suggested very low toxicity in the three cells line used (3T3/NIH, HepG2 and Caco-2), making this compound suitable for biological applications. Furthermore, to evaluate the genotoxicity of **1**·**4PF_6_** in the three cell lines (3T3/NIH, HepG2 and Caco-2), the Single-Cell Gel Electrophoresis, also known as Comet Assay, was used according to guidelines [53]. For this analysis, cell viability values higher than 70% are required, and a genotoxic effect is evaluated considering the percentage of tail intensity, which refers to the DNA fragmentation. For instance, tail intensity values higher than 10% are considered genotoxic. As a positive control, the alkylating-agent methyl methanesulfonate (MMS 400 mM) was used. Despite the previously reported high toxicity of viologens, mainly methyl viologen for their use as non-selective herbicide [54], the results showed that **1**·**4PF_6_** did not promote a significant formation of DNA fragments within the studied concentrations and cell lines tested. This means that below the IC_50_ values no genotoxicity was observed, confirming the capabilities of these gemini-type amphiphilic bipyridinium salts as promising agents for drug encapsulation and further drug release. 

## 4. Conclusions

In conclusion, we present here a versatile supramolecular approach exploiting π-acceptor/π-donor interactions applied to the encapsulation of π-donor neurotransmitters using π-acceptor aromatic bipyridinium salts functionalized silicon-based materials. With this aim, four different bipyridinium hosts (**1**·**4PF_6_**–**4**·**4PF_6_**) were synthesized and characterized to incorporate four neurotransmitters (**D**, **S**, **A** and **NA**). The studies in solution using UV-Vis absorption spectroscopy, fluorescence emission spectroscopy and HPLC demonstrated the ability of bipyridinium hosts to form stable supramolecular π-acceptor/π-donor complexes with **D**, **S**, **A** and **NA**. Moreover, **1**·**4PF_6_**–**3**·**4PF_6_** presented a higher incorporation of **S**, whereas **A** was selectively incorporated by **4**·**4PF_6_**. After the solution studies, the immobilization of the **1**·**4PF_6_**–**4**·**4PF_6_** bipyridinium hosts and incorporation of the neurotransmitters on polysilicon surfaces were accomplished, corroborating each step by contact angle measurements. For the functionalization of polysilicon microparticles, the bipyridinium **1**·**4PF_6_** and **4**·**4PF_6_** and neurotransmitter **A** were selected. The quantification of **A** encapsulated on the microparticles was assessed by HPLC. Functionalized **4**·**4PF_6_** microparticles presented higher encapsulation of **A** than those functionalized with **1**·**4PF_6_**, being 7.77 × 10^−8^ and 4.28 × 10^−8^ μmol/particle, respectively. Moreover, the release studies in the presence of ascorbic acid indicated similar values of **A** released per particle for **1**·**4PF_6_** and **4**·**4PF_6_**, being 5.0 × 10^−9^ and 4.7 × 10^−9^ μmol/particles, respectively. However, with the non-covalent functionalization of microparticles a higher release of the **A** encapsulated was obtained. Finally, cytotoxicity assays of **1**·**4PF_6_** indicated that this bipyridinium host was neither cytotoxic or genotoxic to the cell lines studied 3T3/NIH, HepG2 and Caco-2 at the maximum concentration tested of 500 μg/mL, and therefore a possible desorption of the compound over time from the surfaces will not affect the biocompatibility of the system.

## Figures and Tables

**Table 1:** Contact angles measurements with the corresponding standard deviation of polysilicon surfaces functionalized with **1·4PF_6_**–**4·4PF_6_**, and subsequent incorporation of neurotransmitters **D**, **S**, **A** and **NA**, and control experiments.

Average Contact Angles (θ) ± Standard Deviation (SD) (°)									
Polysilicon Surfaces					44 ± 3				
After Piranha					16 ± 2				
After Basic Treatment					10 ± 1				
**1·4PF_6_**		**2·4PF_6_**		**3·4PF_6_**		**4·4PF_6_**		**Controls**	
**Surface**	**θ ± SD**	**Surface**	**θ ± SD**	**Surface**	**θ ± SD**	**Surface**	**θ ± SD**	**Surface**	**θ ± SD**
1·4PF_6_	57 ± 1	2·4PF_6_	66 ± 1	3·4PF_6_	89± 1	4·4PF_6_	55 ± 4	-	
1·4PF_6_:D	48 ± 1	2·4PF_6_:D	57 ± 1	3·4PF_6_:D	73 ± 2	4·4PF_6_:D	44 ± 3	D	31 ± 3
1·4PF_6_:S	44 ± 1	2·4PF_6_:S	55 ± 1	3·4PF_6_:S	69 ± 2	4·4PF_6_:S	45 ± 2	S	32 ± 1
1·4PF_6_:A	40 ± 1	2·4PF_6_:A	50 ± 2	3·4PF_6_:A	70 ± 1	4·4PF_6_:A	48 ± 2	A	30 ± 1
1·4PF_6_:NA	43 ± 1	2·4PF_6_:NA	52 ± 2	3·4PF_6_:NA	66 ± 1	4·4PF_6_:NA	43 ± 1	NA	30 ± 3

N_3_-SAM = 78 ± 1.

## Supplementary Materials

The following are available online at https://www.mdpi.com/1999-4923/12/8/724/s1, Figures S1–S21: Synthesis of **2·4PF_6_** and **5**, and characterization of **1·4PF_6_**–**4·4PF_6_** and **5**, Figure S22: UV-Vis absorption spectra of **1****·****4PF_6_**, **4****·****4PF_6_**, **D**, **S**, **A** and **NA**, Figure S23: Fluorescence spectra of **1****·****4PF_6_**, **4****·****4PF_6_**, **D**, **S**, **A** and **NA** and their corresponding complexes, Figure S24: Size distribution study of microparticles, Figure S25: Calibration curve of **A** by HPLC, Equation S(1): Protocol for particles counting using a Neubauer chamber, Figure S26: HPLC-Fluorescence chromatograms of initial and final solutions of **A**, Table S1: Values of retention time (RT) and Area under the curve (AUC) extracted from the chromatograms at Figure S26, Table S2: Quantification of **A** encapsulated in **1****·****4PF_6_** and **4****·****4PF_6_** functionalized microparticles, Figure S27: UV-Vis solution studies of complex formation (**4****·****4PF_6_:A**) and disassembly using ascorbic acid, Figure S28: HPLC solution studies of complex formation (**4****·****4PF_6_:A**) and disassembly using ascorbic acid, Figure S29: HPLC-UV-Vis absorption spectra of complex formation (**1****·****4PF_6_:A** and **4****·****4PF_6_:A**) and disassembly, Figure S30: HPLC-Fluorescence chromatograms of release solutions (T1 and T2) of **A** after adding ascorbic acid to the functionalized microparticles, Table S3: Values of retention time (RT) and Area under the curve (AUC) extracted from the chromatograms at Figure S30, Table S4: Quantification of **A** released in **1****·****4PF_6_** and **4****·****4PF_6_** functionalized microparticles by using ascorbic acid, Figure S31: Stability studies of **A** in presence of ascorbic acid, Table S5: Results obtained for cell viability and genotoxicity results for **1****·****4PF_6_** in cell line 3T3/NIH, Table S6: Results obtained for cell viability and genotoxicity results for **1****·****4PF_6_** in cell line HepG2, Table S7: Results obtained for cell viability and genotoxicity results for **1****·****4PF_6_** in cell line Caco-2 and Table S8: Values of the IC_50_ determined for the compound **1****·****4PF_6_** tested in the three different cell lines (3T3/NIH, HepG2 and Caco-2).
