# Supplementary material for: π-Donor/π-Acceptor Interactions for the Encapsulation of Neurotransmitters on Functionalized Polysilicon-Based Microparticles
Source: Pharmaceutics. 2020 Aug 1;12(8):724. doi: 10.3390/pharmaceutics12080724 (PMC7463889; doi:10.3390/pharmaceutics12080724)
Supplement: Supplementary file 1 [file pharmaceutics-12-00724-s001.pdf]

pharmaceutics

Supplementary Materials: π-Donor/π-Acceptor Interactions for the Encapsulation of Neurotransmitters on Functionalized Polysilicon-Based Microparticles

Sandra Giraldo, María E. Alea-Reyes, David Limón, Asensio González, Marta Duch, José A. Plaza, David Ramos-López, Joaquín de Lapuente, Arántzazu González-Campo * and Lluïsa Pérez-García

1. Synthesis and Characterization of Bipyridinium Salts

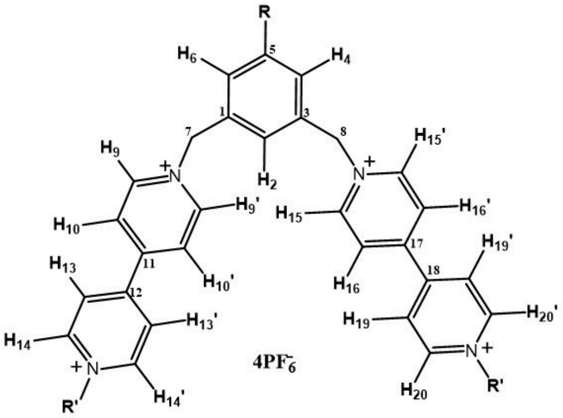

1·4PF$_6$: R = H, R' = CH_3
2·4PF$_6$: R = H, R' = CH_2Bz
3·4PF$_6$: R = H, R' = $C_{16}H_{33}$
4·4PF$_6$: R = HC≡CCH$_2$O, R' = CH_3
5 : R = H, R' = /

Figure S1. Numbering of the hydrogen and carbon atoms in the structures of **1·4PF$_6$**–**4·4PF$_6$** and **5**.

*1.1. Characterization of 1,3-Bis(1′-methyl-4,4′-bipyridiniummethylene) Benzene-Tetrakis (Hexafluorophosphate) (**1·4PF₆**)*

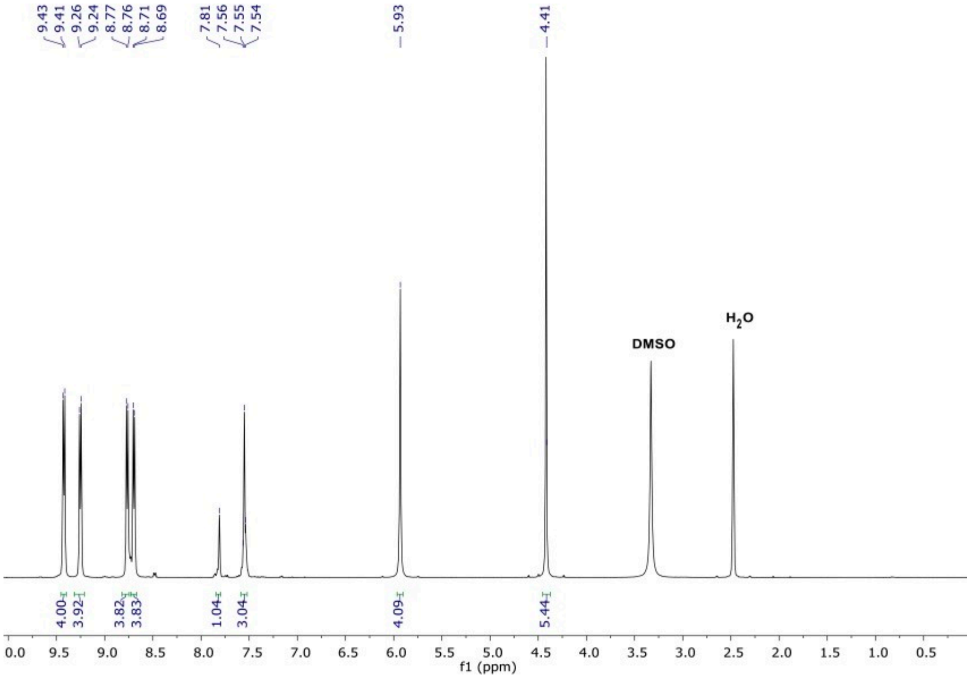

Figure S2. ^{1}H NMR (400 MHz) spectrum of **1·4PF$_6$** in $(CD_3)_2SO$.

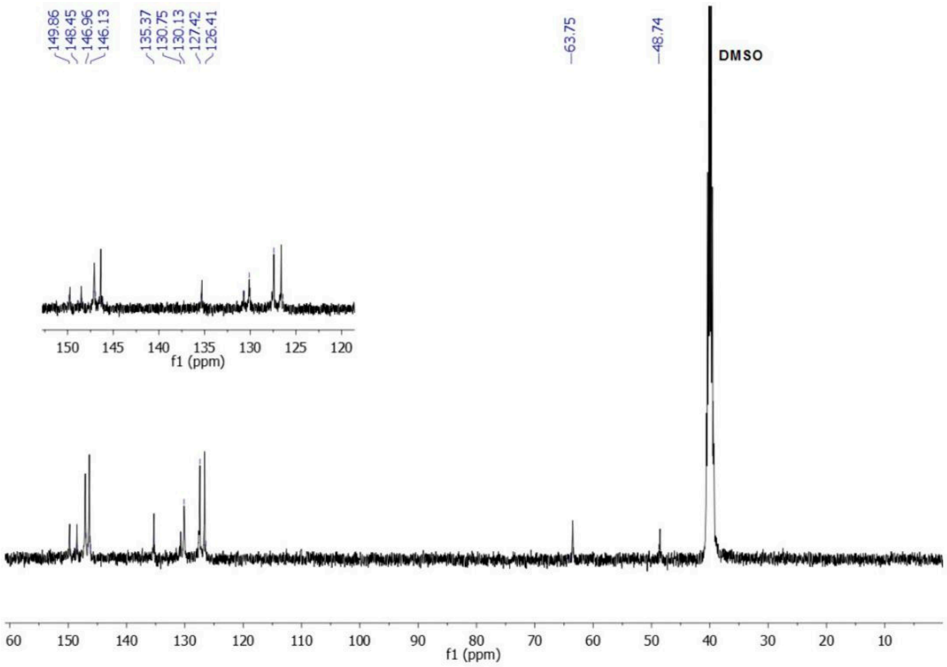

Figure S3. ^{13}C NMR (400 MHz) spectrum of **1·4PF$_6$** in $(CD_3)_2SO$.

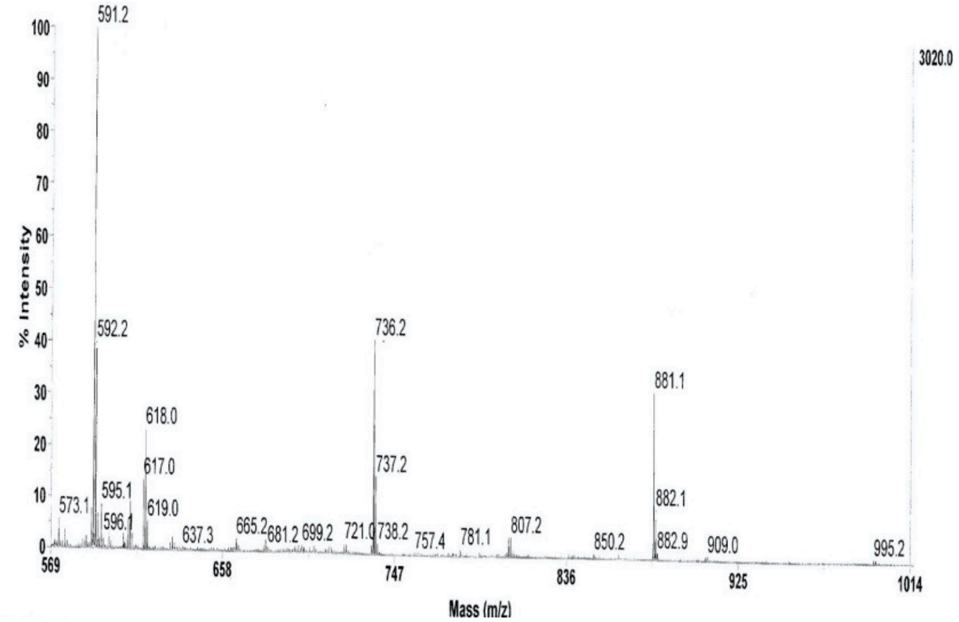

Figure S4. MALDI-TOF-MS (m/z) spectrum of **1·4PF₆** with matrix DHB.

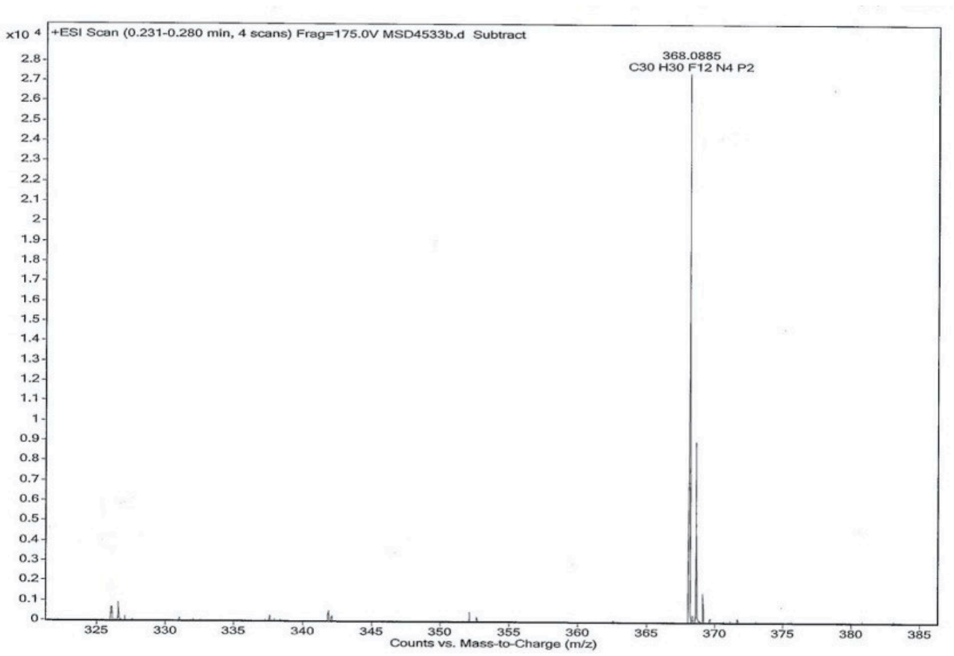

Figure S5. HMRS-ESI (m/z) spectrum of **1·4PF₆**.

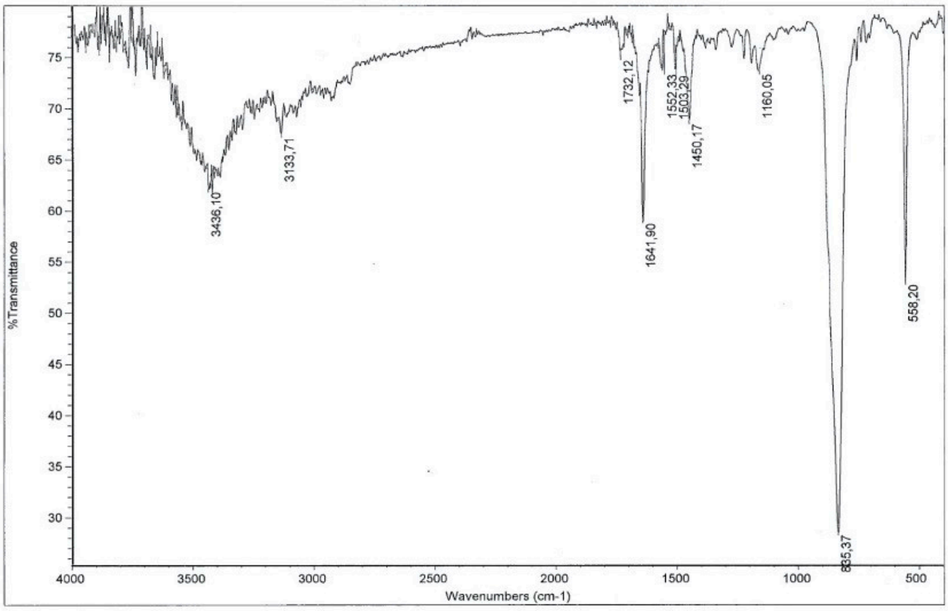

Figure S6. FT-IR transmittance spectrum of **1·4PF$_6$** in KBr.

*1.2. Synthesis and Characterization of 1,3-Bis(1′-dibenzyl-4,4′bipyridinium methylene) Benzene Tetrakis (Hexafluorophosphate) (**2·4PF$_6$**):*

A solution of **5** (0.7 g, 1.1 mmol) in CH_3NO_2 (30 mL) was added to a solution of benzyl bromide (1.2 g, 6.9 mmol) in CH_3NO_2 (4 mL). The mixture was heated at 80 °C for 3 h. After cooling down to room temperature, the solvent was evaporated in vacuum. The yellow residue was then dissolved in H_2O (10 mL), and a saturated aqueous solution of NH_4PF_6 (1.1 g, 6.8 mmol, 3 mL) was added until no further precipitation was observed. The suspension was filtered off and the white solid was washed with H_2O (30 mL), filtered off and dried under vacuum to afford **2·4PF$_6$** (0.9 g, 85%). mp = 260 °C (Lit. [1]); ^{1}H NMR (400 MHz, $(CD_3)_2SO$, 25 °C): δ 9.48 (d, *J* = 4 Hz, 4H, H-9, 9', 15, 15'), 9.42 (d, *J* = 4 Hz, 4H, H-14, 14', 20, 20'), 8.73 (dd, *J* = 4 Hz, *J* = 4 Hz, 8H, H-10, 10', 16, 16', 13, 13', 19, 19'), 7.82 (s; 1H, H-2); 7.59 (d, *J* = 4 Hz ,4H, H-Bz), 7.57 (d, *J* = 4 Hz, 4H, H-Bz), 7.54 (s; 2H, H-Bz); 7.45 (m;3H, H-4, 5, 6), 5.92 (s; 8H, H-7, 8, -*CH*$_2$-Bz). MALDI-TOF–MS m/z: 1033.1 m/z (3%) $[M\text{-}1PF_6]^+$, 888.1 (40%) $[M\text{-}2PF_6]^+$, 743.2 (38%) $[M\text{-}3PF_6]^+$, 598.2 (100%) $[M\text{-}4PF_6]^+$, 351.1 (45%) $[M\text{-}(C_{17}H_{15}N_2(4PF_6))]^+$. HMRS (ESI) m/z: $(C_{42}H_{38}F_{12}N_4P_2)^{2+}$ calc 444.13 found 444.11. IR spectrum (KBr, cm^{-1}): 3137-3068 ν(C–H) (alkane), 1638 ν(C=N) and 834 δ(C=C).

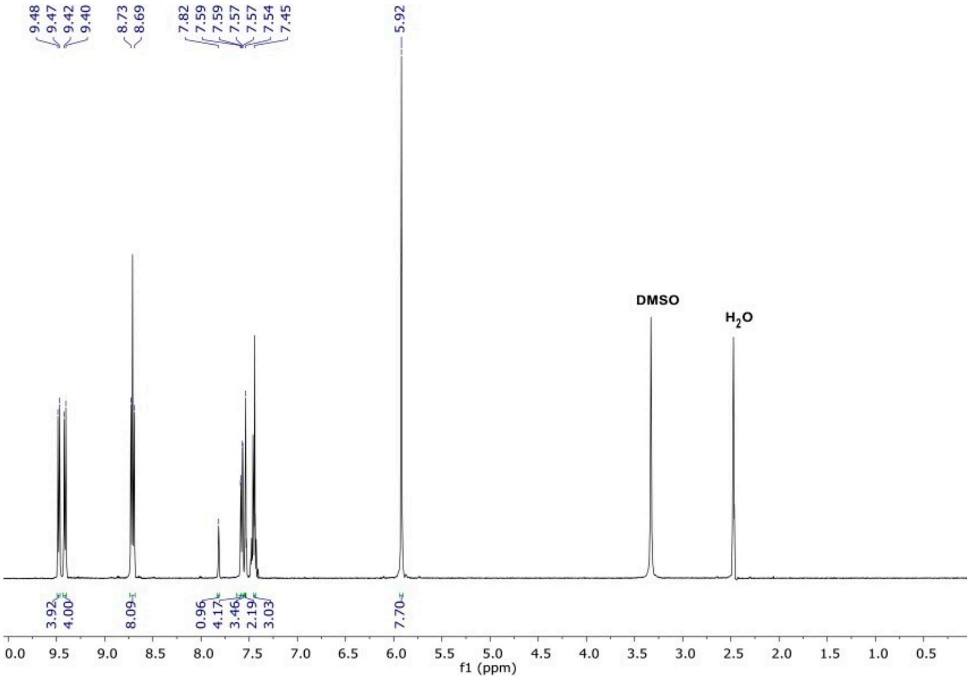

Figure S7. ^{1}H NMR (400 MHz) spectrum of **2·4PF$_6$** in $(CD_3)_2SO$.

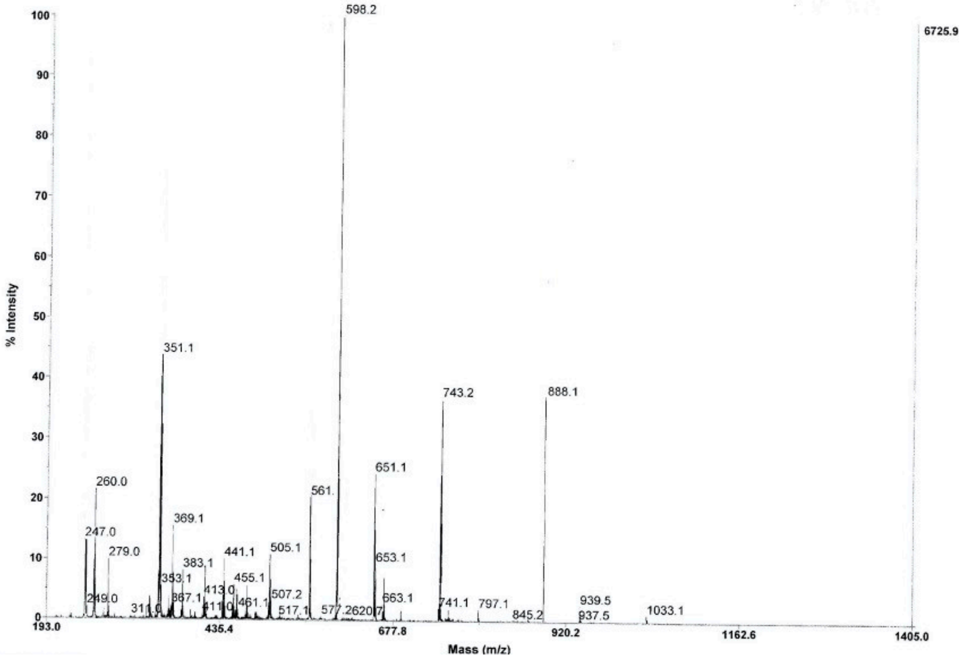

Figure S8. MALDI-TOF-MS (m/z) spectrum of **2·4PF$_6$** with matrix DHB.

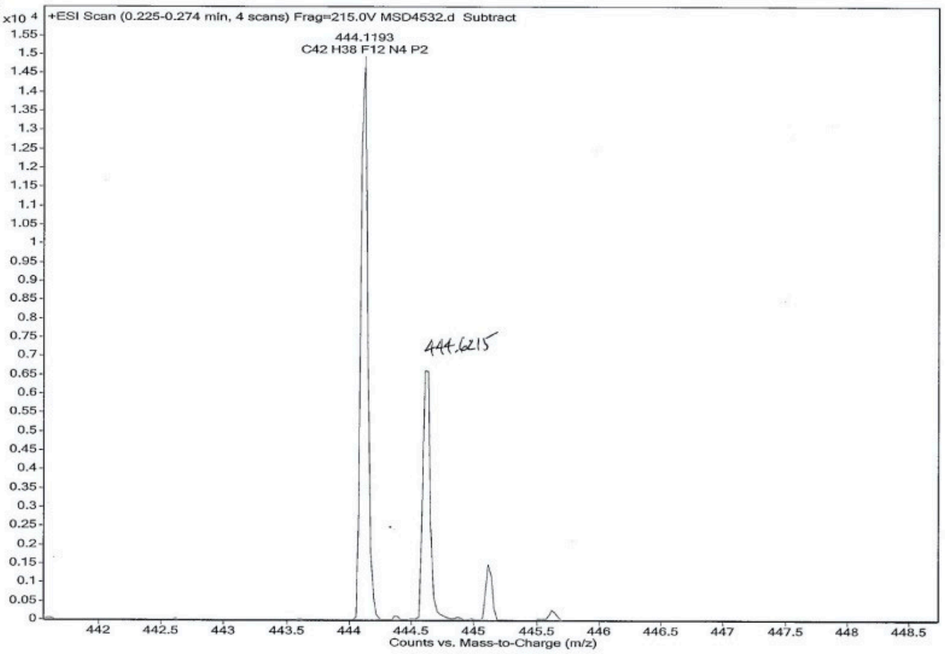

Figure S9. HMRS-ESI (m/z) spectrum of **2·4PF6**.

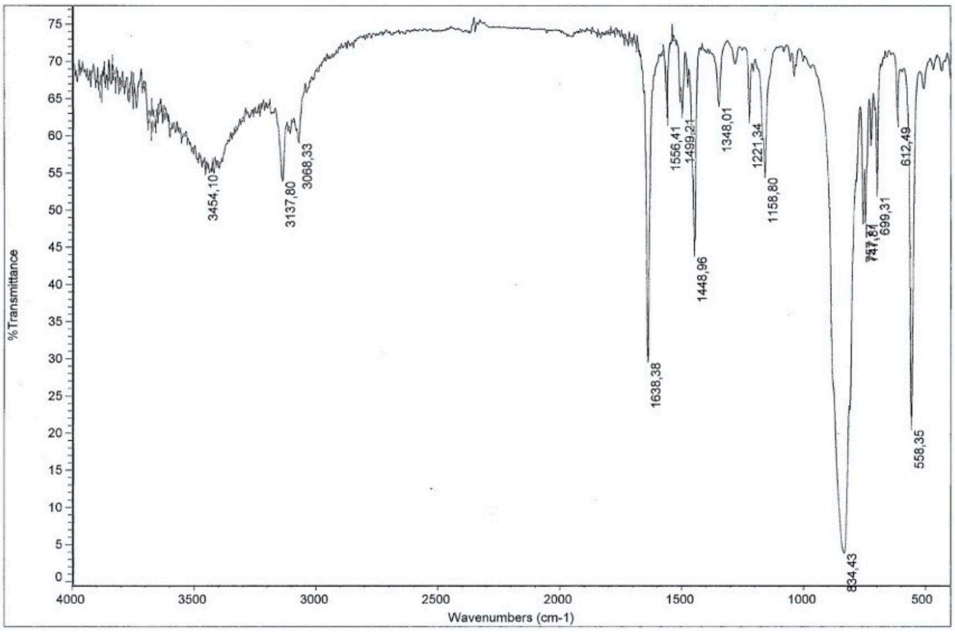

Figure S10. FT-IR transmittance spectrum of **2·4PF6** in KBr.

1.3. Characterization of 1,3-Bis(1′-hexadecyl-4,4′-bipyridiniummethylene) Benzene-Tetrakis (Hexafluorophosphate) (3·4PF₆)

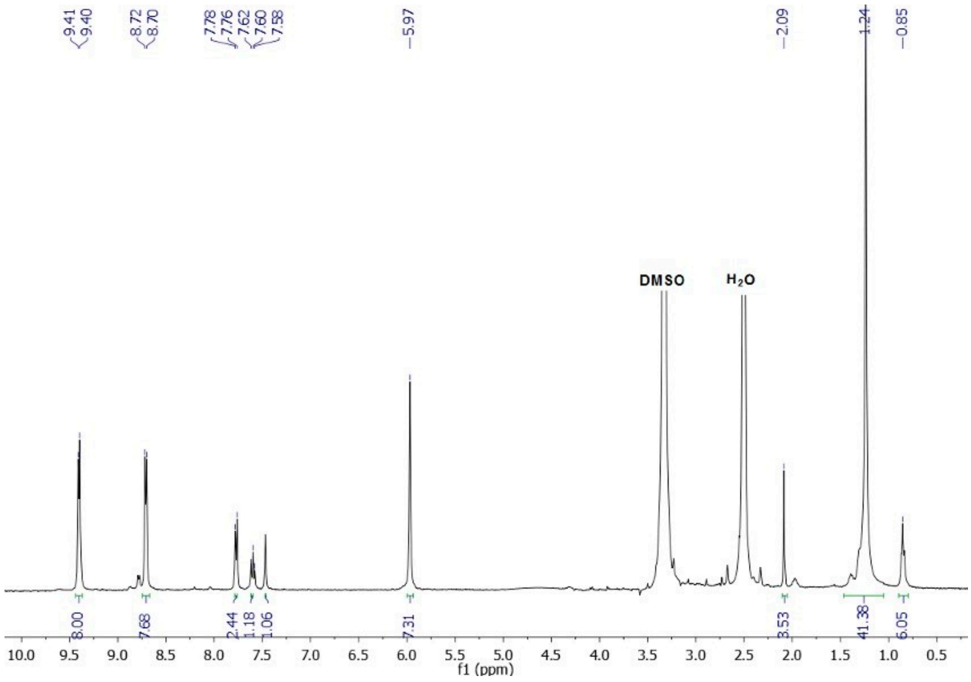

Figure S11. ^{1}H NMR (400 MHz) spectrum of **3·4PF$_6$** in $(CD_3)_2SO$.

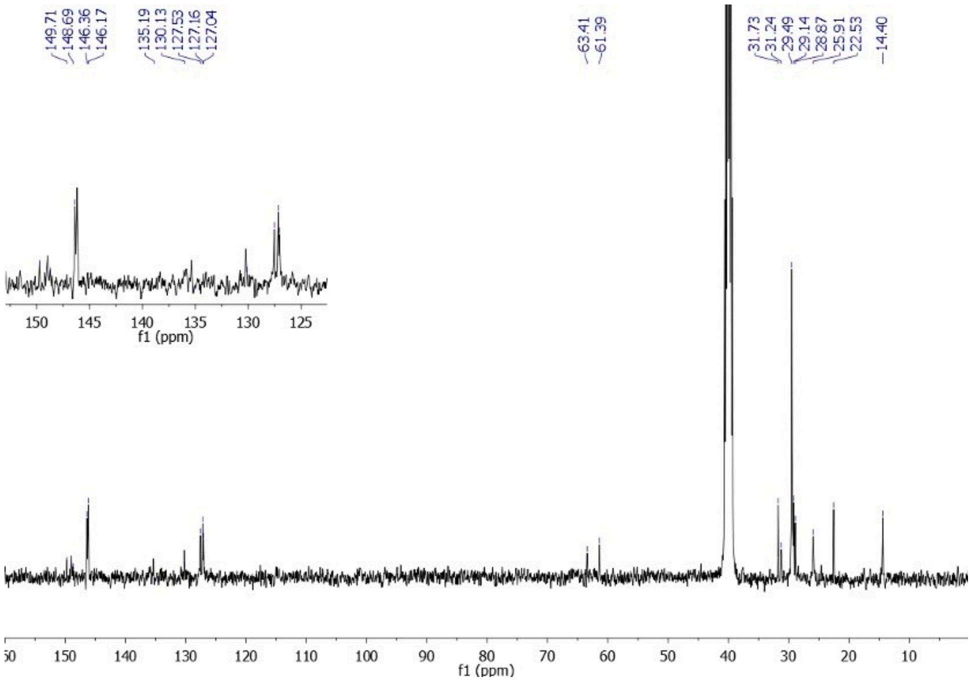

Figure S12. ^{13}C NMR (400 MHz) spectrum of **3·4PF$_6$** in $(CD_3)_2SO$.

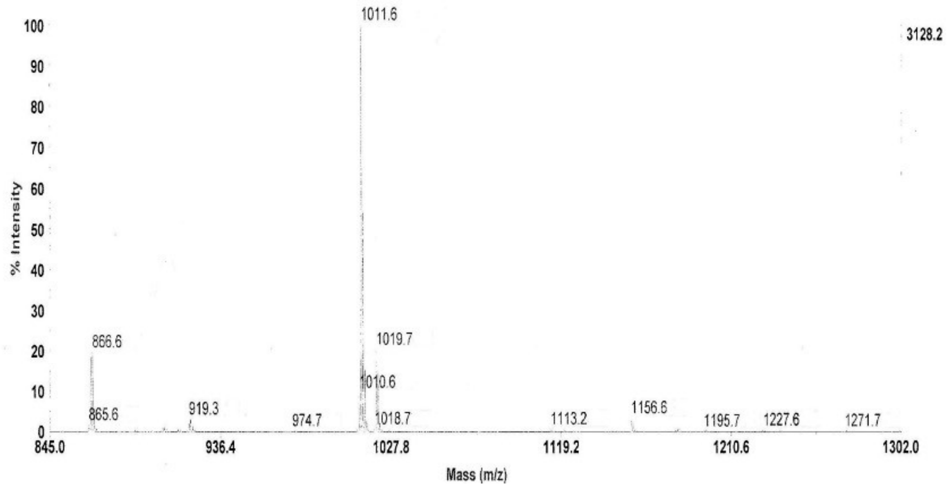

Figure S13. MALDI-TOF-MS (m/z) spectrum of **3·4PF**$_6$ with matrix DHB.

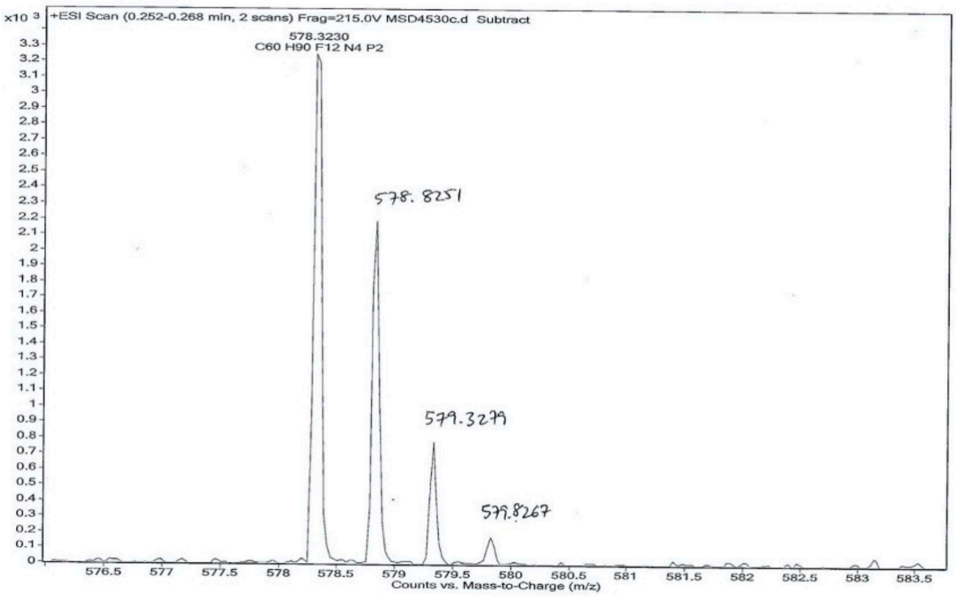

Figure S14. HMRS-ESI (m/z) spectrum of **3·4PF**$_6$.

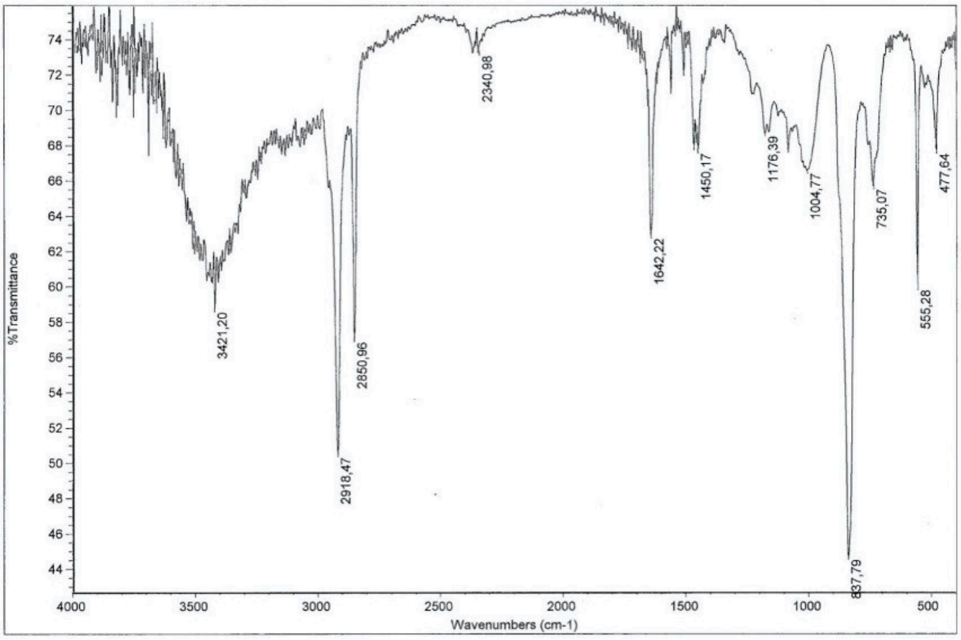

Figure S15. FT-IR transmittance spectrum of **3·4PF₆** in KBr.

1.4. Characterization of 1,3-Bis(1′-methyl-4,4′-bipyridiniummethylene)-5-propargyloxy-benzene-tetrakis (Hexafluorophosphate) (4·4PF₆)

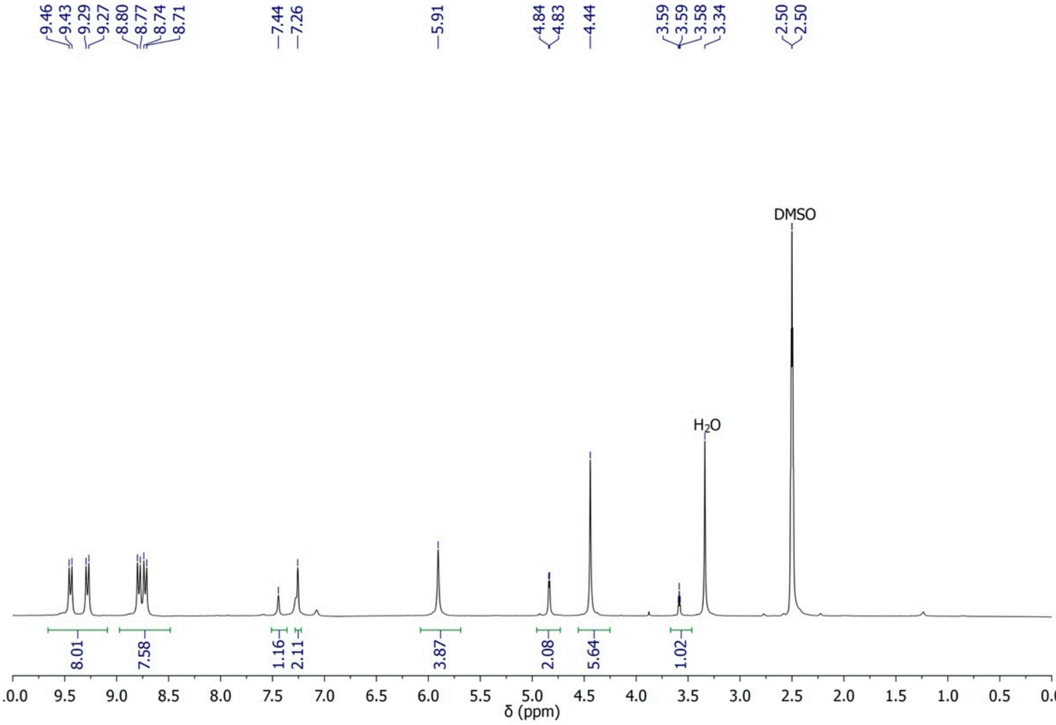

Figure S16. ^{1}H NMR (250 MHz) spectrum of **4·4PF₆** in DMSO.

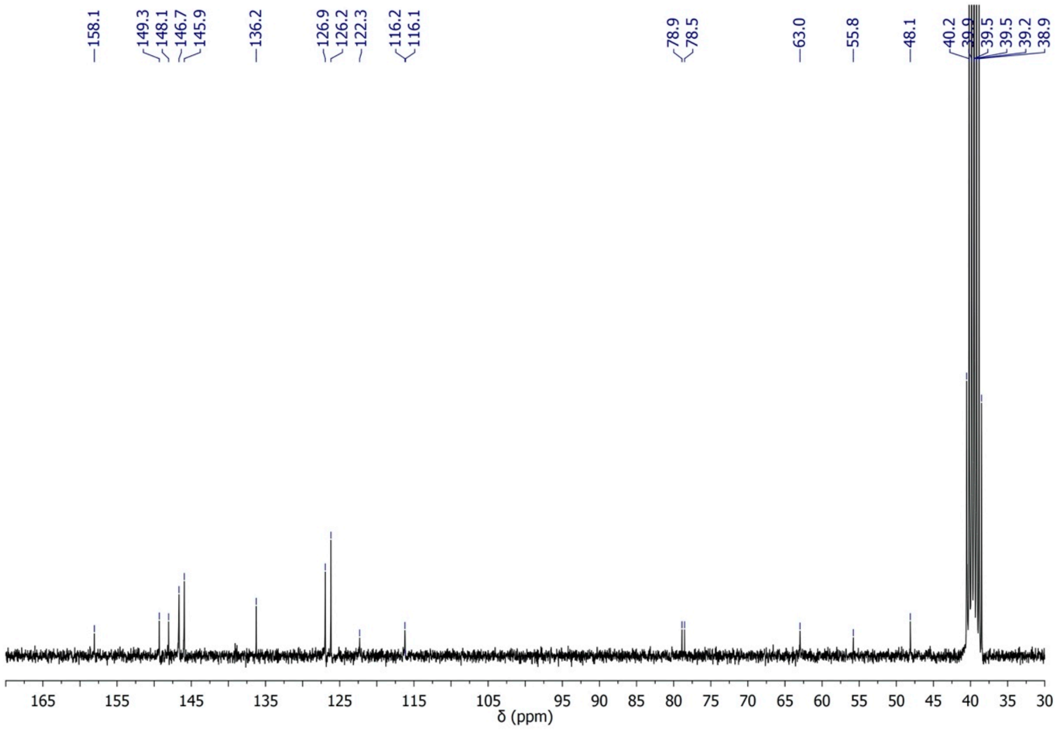

Figure S17. ^{13}C NMR (250 MHz) spectrum of **4·4PF$_6$** in DMSO.

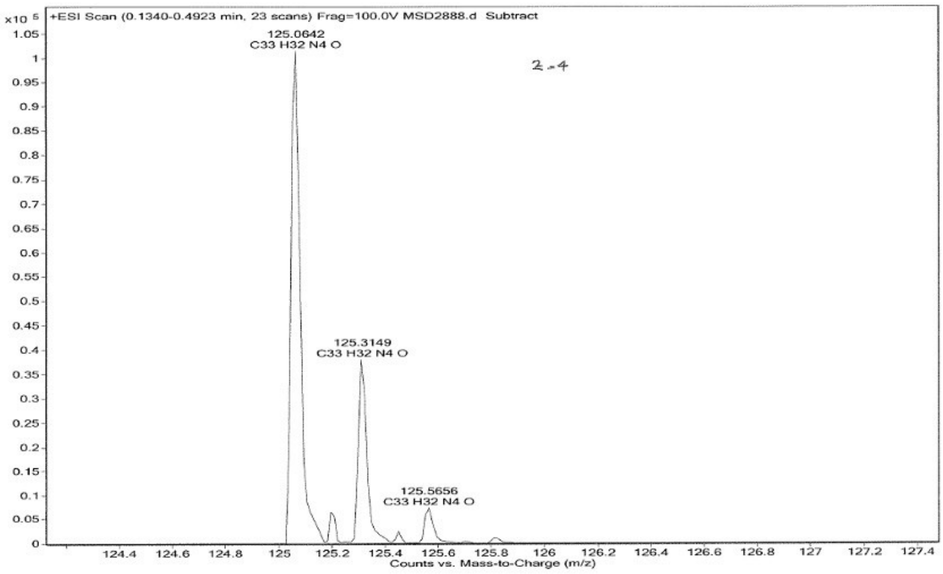

Figure S18. HMRS-ESI (m/z) spectrum of **4·4PF$_6$**.

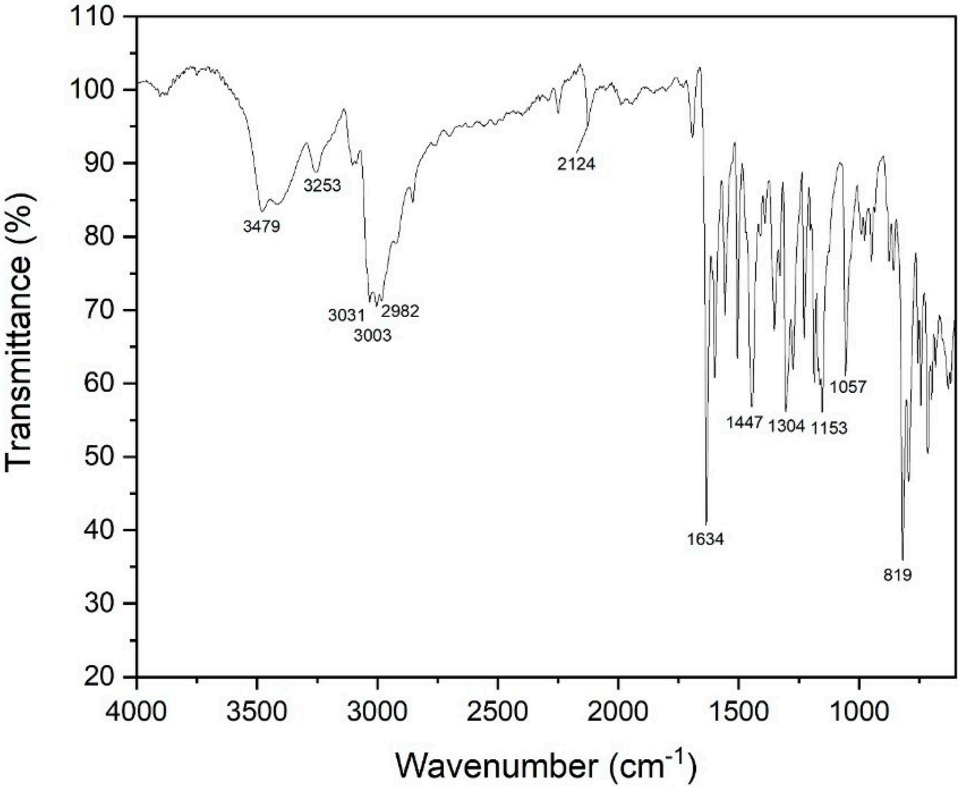

Figure S19. FT-IR (ATR) transmittance spectrum of **4·4PF$_6$**.

1.5. Synthesis and Characterization of 1,3-Bis (phenylene-4,4′- bipyridinium methylene) Benzene-Di(bromide) (5)

A solution of 4,4'-bipyridine (3.1 g, 19.9 mmol) in dry CH_3CN (30 mL) was added dropwise during 30 min to a solution of 1,3-bis(bromomethyl)benzene (2.2 g, 8.3 mmol) in dry CH_3CN (40 mL), which was heated under reflux. Heating was continued for 2 h. After cooling down to room temperature, the yellow precipitate was filtered off and washed with CH_3CN (10 mL) and dried under vacuum to afford **5** (4.7 g, 98%). mp = 260 °C (Refs:~~Lit.~~ [1,2]); ^{1}H NMR (300 MHz, $(CD_3)_2SO$, 25 °C): δ 9.35 (d, *J* = 6 Hz, 4H, H-9, 9', 15, 15'), 8.88 (d, *J* = 6 Hz, 4H, H-10, 10', 16, 16'), 8.68 (d, J = 6 Hz, 4H, H-13, 13', 19, 19'), 8.03 (d, *J* = 6 Hz, 4H, H-14, 14', 20, 20'); 7.76 (s; 1H, H-2), 7.58 (m; 3H, H-4, 5, 6), 5.91 (s; 4H, H-7, 8). MALDI-TOF–MS m/z: 415.1 (90%) $[M\text{-}2Br\text{-}1H]^+$, 341.0 (45%) $[M\text{-}(C_{10}H_8BrN_2)]^+$, 261.1 (13%) $[M\text{-}(C_{10}H_8Br_2N_2)]^+$.

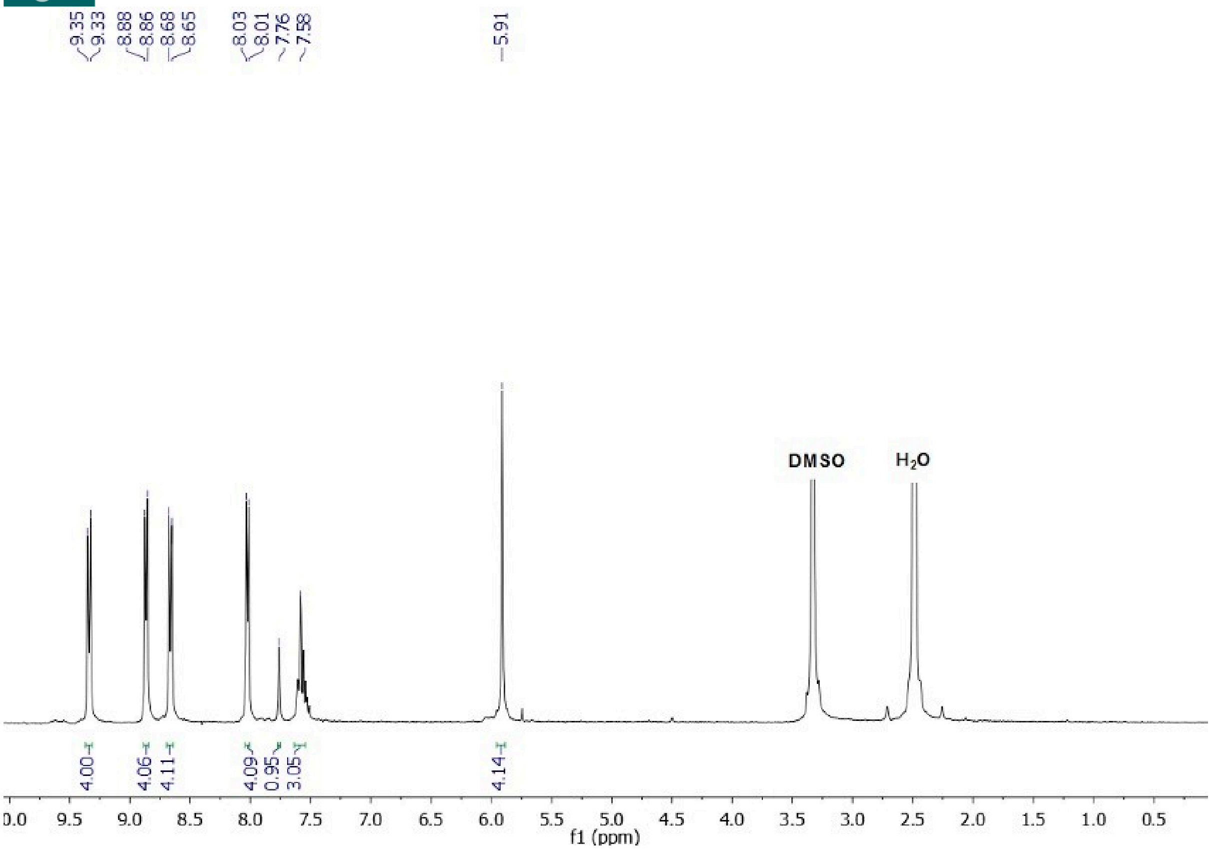

Figure S20. ^{1}H NMR (300 MHz) spectrum of **5** in $(CD_3)_2SO$.

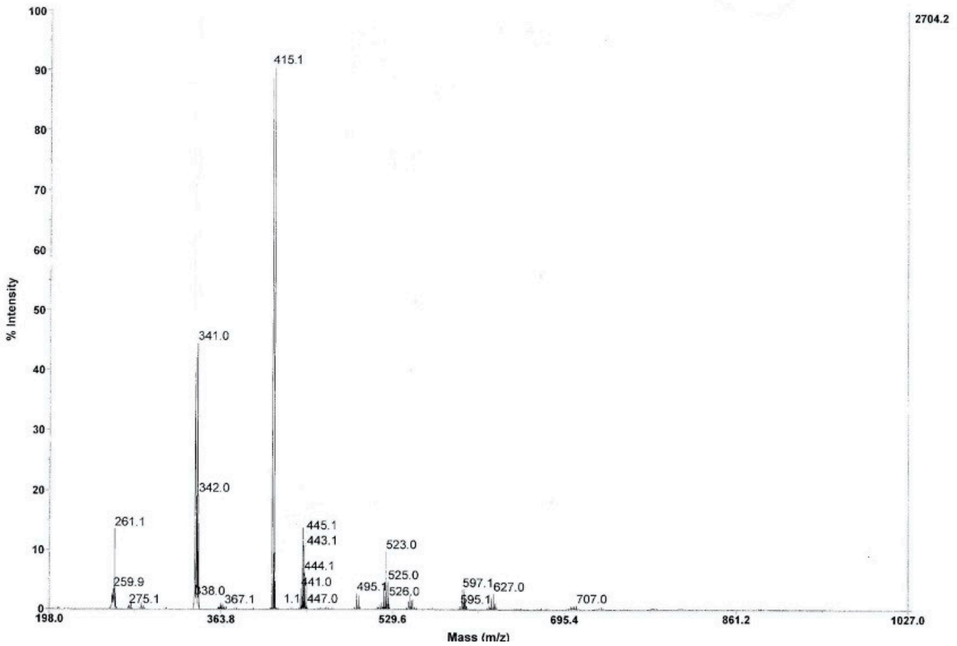

Figure S21. MALDI-TOF-MS (m/z) spectrum of **5** with matrix DHB.

2. UV-Vis Absorption Spectra of 1·4PF6, 4·4PF6 and Neurotransmitters: D, S, A and NA

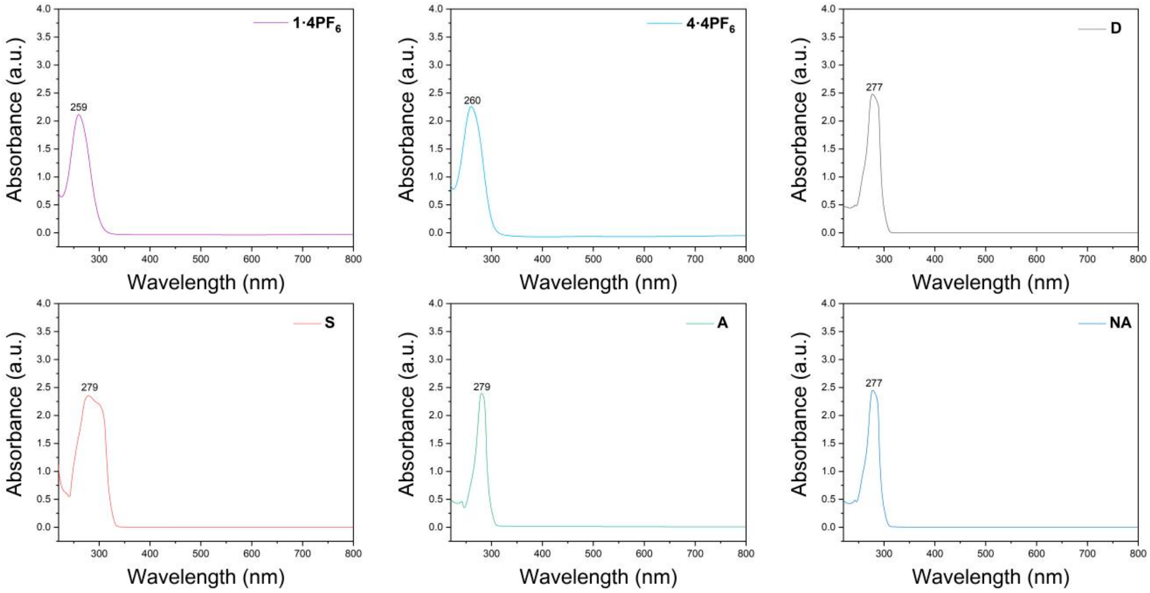

Figure S22. UV-Vis absorption spectra of **1·4PF6**, **4·4PF6**, **D**, **S**, **A** and **NA** in DMSO: H_2O (4:1) at 1 mM.

3. Fluorescence Spectra of 1·4PF6, 4·4PF6 and Neurotransmitters: D, S, A and NA and Their Corresponding Complexes

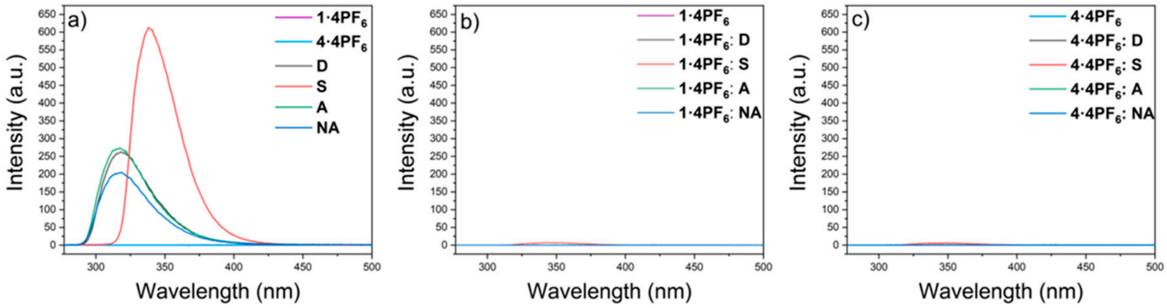

Figure S23. Fluorescence emission spectra of: (a) **1·4PF6**, **4·4PF6**, **D**, **S**, **A** and **NA**, respectively; (b) Complex formation of **1·4PF6** with **D**, **S**, **A** and **NA**; (c) Complex formation of **4·4PF6** with **D**, **S**, **A** and **NA**; All spectra were recorded at 2 mM in DMSO: H_2O (4:1) at λ_{exc} = 259, 260, 277, 279, 279, 279 nm for **1·4PF6**, **4·4PF6**, **D**, **S**, **A** and **NA**, respectively.

4. Size Distribution Histograms of Microparticles

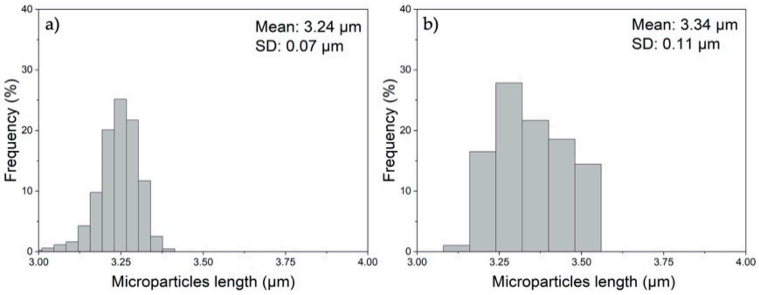

Figure S1. Particle size distribution histograms of: (**a**) Poly-Surfs analysing SEM images and 870 microparticles; (**b**) Poly-Si μPs suspended in solution analysing optical microscope images and 100 microparticles.

5. Calibration Curve of A by HPLC Determination

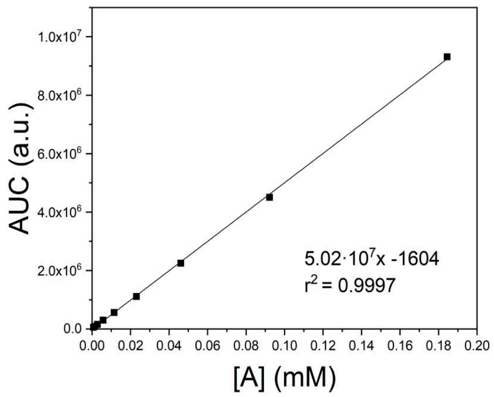

Figure S25. Calibration curve of **A** by HPLC coupled to a fluorescence detector at λ_{exc} = 280 nm and λ_{em} = 315 nm. The concentrations of **A** were: 0.18, 0.09, 0.04, 0.02, 0.01, 5.8 × 10^{-3}, 2.9·10^{-3}, 1.4 × 10^{-3}, 7.2 × 10^{-4} mM, using deionized H_2O as solvent and taking the area of the fluorescence peak at each concentration.

6. Particles Counting Protocol

Equation S(1) used for particles counting in a Neubauer chamber [3] by ImageJ software [4].

$$N(microparticles) = \frac{X \times 10000}{N°square \times dilution} \quad (1)$$

X = microparticles counted by ImageJ (Analyze particles) (2)

7. Fluorescence Chromatograms for Quantification of A Encapsulated in Poly-Si μPs by HPLC

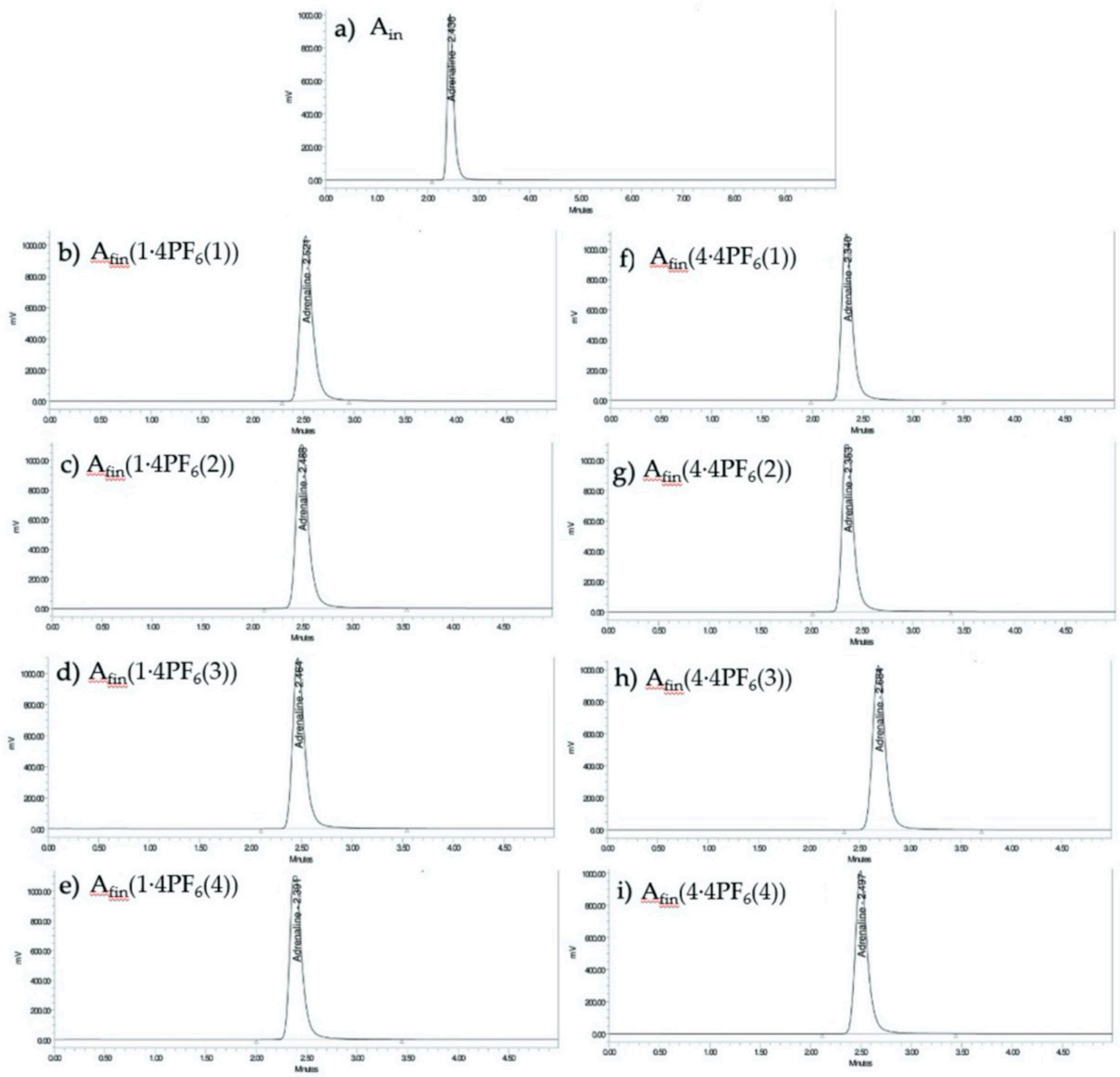

Figure S26. Fluorescence chromatograms of initial (A_{in}) and final solutions (A_{fin}) of **A** from respective 1–4 microtubes, before (**a**) and after the incubation with **1·4PF$_6$** functionalized microparticles (**b–e**) or **4·4PF$_6$** functionalized microparticles (**f–i**), by HPLC determination at λ_{exc} = 280 nm and λ_{em} = 315 nm.

Table S1. Values of retention time (**RT**) and area under the curve (**AUC**) extracted from the chromatograms at Figure S25.

		RT	AUC (a.u)
	**A$_{in}$**	2.438	10712528
**A$_{fin}$**	**(1·4PF$_6$(1))**	2.521	8959754
**A$_{fin}$**	**(1·4PF$_6$(2))**	2.488	9778307
**A$_{fin}$**	**(1·4PF$_6$(3))**	2.464	9254002
**A$_{fin}$**	**(1·4PF$_6$(4))**	2.391	9413136
**A$_{fin}$**	**(4·4PF$_6$(1))**	2.340	8359152
**A$_{fin}$**	**(4·4PF$_6$(2))**	2.353	8892739
**A$_{fin}$**	**(4·4PF$_6$(3))**	2.684	9189019
**A$_{fin}$**	**(4·4PF$_6$(4))**	2.497	8551537

pharmaceutics

Table S2. Quantification of **A** encapsulated (**A$_{encap.}$**) in **1·4PF$_6$** and **4·4PF$_6$** functionalized microparticles (µP).

	**A$_{in}$ (µmol)**	**A$_{fin}$ (µmol)**	**A$_{encap.}$ (µmol)**	**Nº Particles**	**A$_{encap.}$/Particle (µmol)**
**µP 1·4PF$_6$**	1.71 ± 0.02	1.49 ± 0.01	0.22 ± 0.01	5,064,000 ± 3,275,131	4.28 × 10^{-8} ± 0.01
**µP 4·4PF$_6$**	1.71 ± 0.02	1.39 ± 0.01	0.32 ± 0.01	4,027,000 ± 1,927,456	7.77 × 10^{-8} ± 0.01

8. UV-Vis solution Studies of Complex Formation (4·4PF$_6$:A) and Disassembly Using Ascorbic Acid

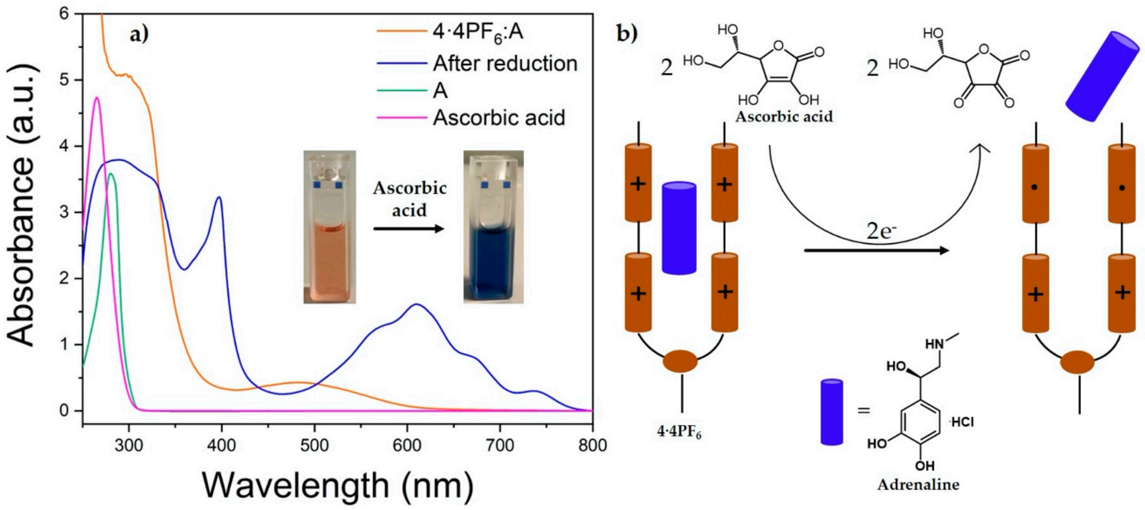

Figure S2. (**a**) UV-Vis absorption spectra of the complex formation **4·4PF$_6$**: **A** (2 mM: 2 mM) in DMSO: H_2O (4:1) after 24 h (orange line), the spectrum of the same solution after the addition of ascorbic acid (4 mM) (blue line), **A** (2 mM) (green line) and ascorbic acid (4 mM) (pink line); (**b**) Representation of the redox process after reduction of bipyridinium salt using ascorbic acid.

9. HPLC Solution Studies of Complex Formation and Disassembly Using Ascorbic Acid

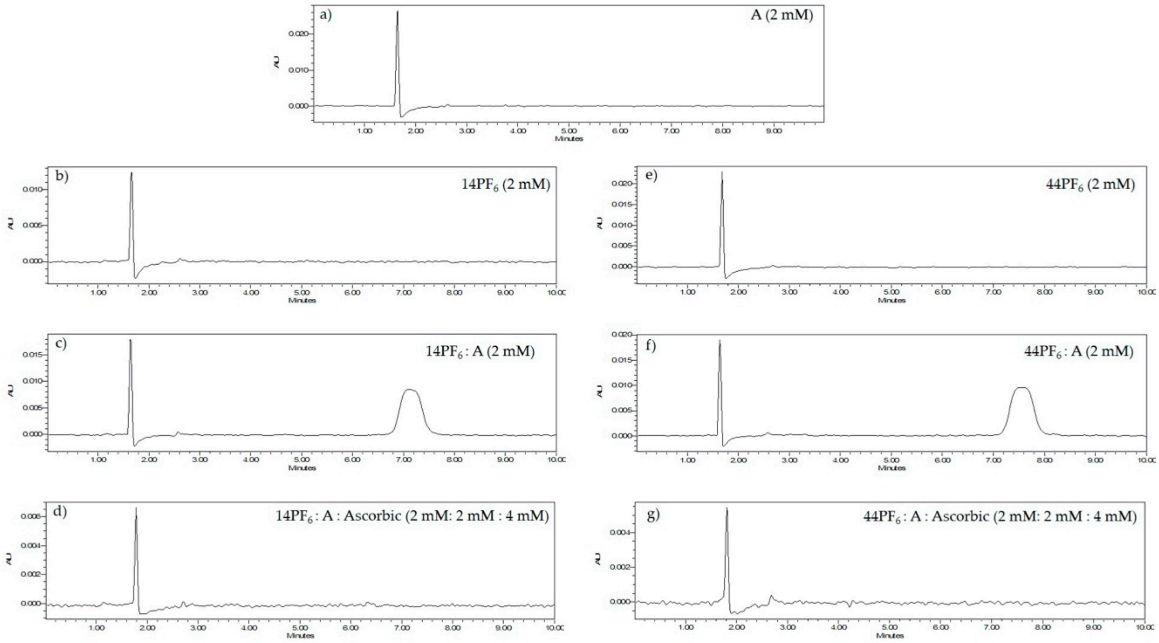

Figure S28. HPLC-UV-Vis chromatograms at absorbance wavelength of 479 nm of: (**a**) **A** (2 mM); (**b**) **1·4PF**$_6$ (2 mM); (**c**) Complex of **1·4PF**$_6$: **A** (2 mM: 2 mM); (**d**) **1·4PF**$_6$: **A**: Ascorbic acid (2 mM:2 mM: 4 mM); (**e**) **4·4PF**$_6$ (2 mM); (**f**) Complex of **4·4PF**$_6$: **A** (2 mM: 2 mM); (**g**) **4·4PF**$_6$: **A**: Ascorbic acid (2 mM: 2 mM:4 mM). All solutions were in DMSO:H_2O (4:1).

10. UV-Vis Absorption Spectra of Complex Formation and Disassembly in Solution by HPLC

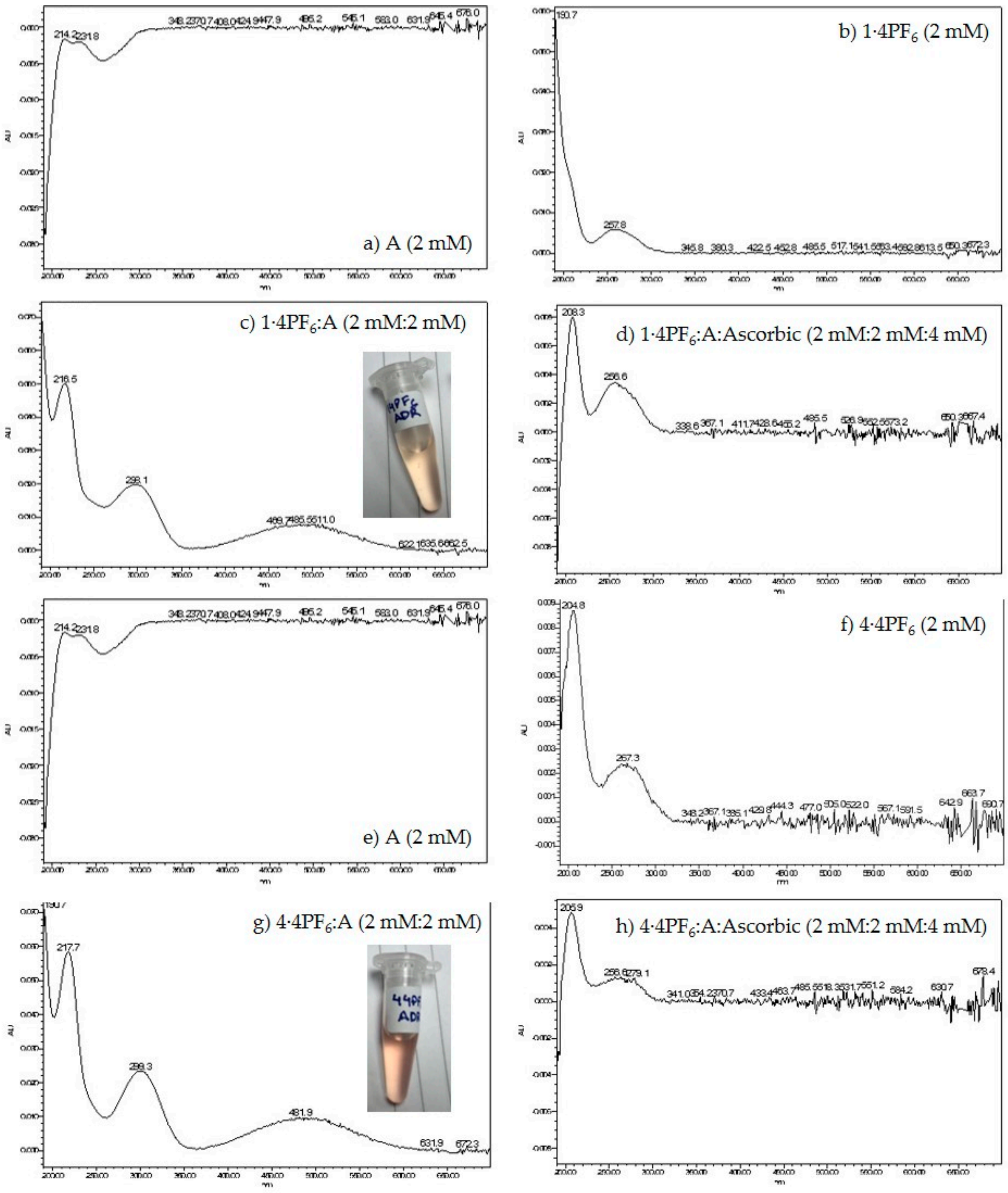

Figure S29. Absorption spectra extracted from the chromatograms at 479 nm from the solution experiments corresponding to retention times at 7.25 min (**1·4PF₆**) and 7.50 min (**4·4PF₆**), obtained by HPLC coupled with a UV-Vis absorption spectroscopy detector.

pharmaceutics MDPI

11. Fluorescence Chromatograms for Quantification of A Released by HPLC

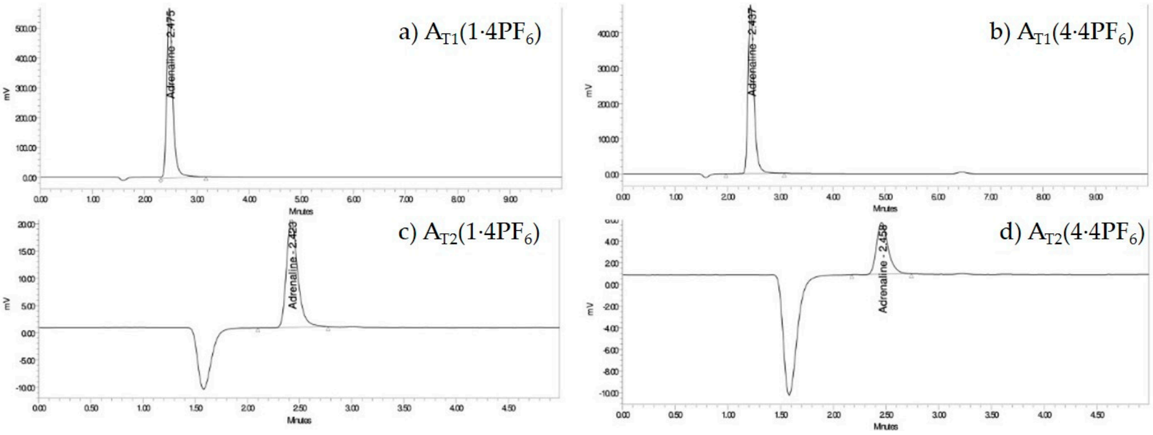

Figure S3. Fluorescence chromatograms of release solutions (T1 and T2) of **A** after the incubation of functionalized microparticles (**1·4PF**$_6$ and **4·4PF**$_6$) with ascorbic acid (4 mM), by HPLC determination at λ_{exc}= 280 nm and λ_{em}= 315 nm.

Table S3. Values of retention time (**RT**) and area under the curve (**AUC**) for functionalized microparticles extracted from the chromatograms at Figure S.29.

	**RT**	**AUC (a.u)**
**A**$_{T1}$ **(1·4PF**$_6$**)**	2.437	3667147
**A**$_{T2}$ **(1·4PF**$_6$**)**	2.423	148759
**A**$_{T1}$ **(4·4PF**$_6$**)**	2.475	4600523
**A**$_{T2}$ **(4·4PF**$_6$**)**	2.458	36665

Table S4. Quantification of **A** released in functionalized microparticles with **1·4PF**$_6$ or **4·4PF**$_6$ using ascorbic acid.

	**A**$_{encap.}$ **(µmol)**	**A**$_{T1}$ **(µmol)**	**A**$_{T2}$ **(µmol)**	**A**$_{released}$ **(µmol)**	**Nº particles**	**A**$_{released}$ **/ particle (µmol)**	**A**$_{released}$ **(%)**
**µP 1·4PF**$_6$	0.22 ± 0.01	0.018 ± 0.01	$6.6{\cdot}10^{-4}$ ± $2{\cdot}10^{-4}$	0.019 ± 0.01	3,807,000 ± 1,546,197	$5.00{\cdot}10^{-9}$ ± 0.02	9 ± 0.01
**µP 4·4PF**$_6$	0.32 ± 0.01	0.015 ± $3{\cdot}10^{-3}$	$2.1{\cdot}10^{-4}$ ± $1{\cdot}10^{-4}$	0.015 ± $3{\cdot}10^{-3}$	3,192,000 ± 892,825	$4.67{\cdot}10^{-9}$ ± $6{\cdot}10^{-3}$	5 ± 0.3

12. Stability Studies of A In Presence of Ascorbic Acid

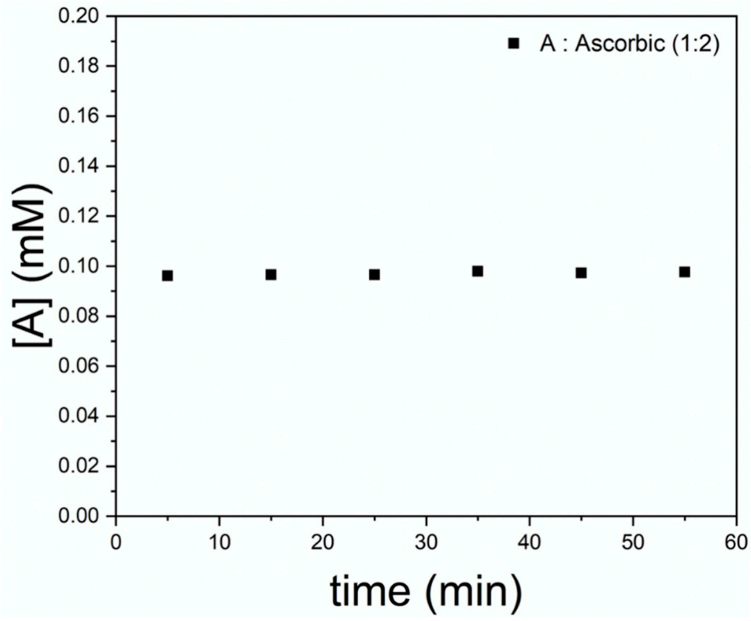

Figure S4. Stability study of **A** in presence of ascorbic acid in ratio **A**: ascorbic (1:2) using deionized H_2O as solvent.

13. Cytotoxic Studies of 1·4PF$_6$

Table S5. Results obtained for Cell viability and genotoxicity results for **1·4PF$_6$** in cell line **3T3/ NIH**, with 500 µg/mL as maximum tested concentration; n/d not determined (viability < 70%).

Compound	Concentration (µg/ mL)	Viability (%) ± SD	Tail moment (%) ± SD
**1·4PF$_6$**	Control medium	100.0 ± 0.2	3.9 ± 4.7
	Control vehicle	63.6 ± 15.2	2.8 ± 3.9
	500.0	46.8 ± 2.3	n/d
	158.1	15.4 ± 1.6	n/d
	50.0	8.7 ± 2.2	n/d
	15.8	79.3 ± 46.4	2.2 ± 1.9
	5.0	150.5 ± 48.4	1.8 ± 1.7
	Positive control	3.1 ± 0.3	51.2 ± 7.1

Table S6. Results obtained for Cell viability and genotoxicity results for **1·4PF$_6$**, in cell line **HepG2**, with 500 mg/mL as maximum tested concentration; n/d not determined (viability < 70%).

Compound	Concentration (µg/ mL)	Viability (%) ± SD	Tail moment (%) ± SD
**1·4PF$_6$**	Control medium	100.0 ± 0.5	3.1 ± 4.2
	Control vehicle	77.4 ± 7.4	2.3 ± 2.8
	500.0	74.7 ± 14.8	23.6 ± 7.0
	158.1	24.1 ± 9.9	38.4 ± 11.4
	50.0	14.1 ± 4.9	n/d
	15.8	35.2 ± 20.3	27.5 ± 21.5
	5.0	107.6 ± 37.5	8.9 ± 14.4
	Positive control	4.0 ± 2.2	35.5 ± 9.6

Table S7. Results obtained for Cell viability and genotoxicity results for **1·4PF$_6$**, in cell line **Caco-2**, with 500 µg/mL as maximum tested concentration; n/d not determined (viability < 70%).

Compound	Concentration (µg/ mL)	Viability (%) ± SD	Tail moment (%) ± SD
**1·4PF$_6$**	Control medium	100.0 ± 0.1	1.5 ± 2.0
	Control vehicle	96.8 ± 8.3	2.4 ± 2.9
	500.0	8.8 ± 3.6	18.4 ± 11.0
	158.1	8.5 ± 3.1	n/d
	50.0	50.5 ± 4.5	18.6 ± 17.1
	15.8	86.9 ± 16.9	4.7 ± 4.5
	5.0	113.7 ± 31.7	4.6 ± 5.1
	Positive control	12.4 ± 2.7	27.1 ± 9.0

Table S8. Values of the IC$_{50}$ determined for the compound **1·4PF$_6$** tested in the three different cell lines with a maximum tested concentration of 500 µg/mL.

Compound	3T3/ NIH		HepG2		Caco-2	
	IC$_{50}$ (µg/mL)	IC$_{50}$ (µM)	IC$_{50}$ (µg/mL)	IC$_{50}$ (µM)	IC$_{50}$ (µg/mL)	IC$_{50}$ (µM)
**1·4PF$_6$**	146	140	152	148	125	122

References

1. Geuder, W.; Hünig, S.; Suchy, A. Single and double bridged viologenes and intramolecular pimerization of their cation radicals. *Tetrahedron* **1986**, *42*, 1665–1677.
2. Ashton, P.R.; Pérez-García, L.; Stoddart, J.F.; Ballardini, R.; Balzani, V.; Credi, A.; Gandolfi, M.T.; Prodi, L.; Venturi, M.; Menzer, S.; et al. Molecular Meccano. 4. The Self-Assembly of [2] Catenanes Incorporating Photoactive and Electroactive π-Extended Systems. *J. Am. Chem. Soc.* **1995**, *117*, 11171–11197.
3. Bastidas, O. Neubauer Chamber Cell Counting Available online: https://www.academia.edu/13150839/Technical_Note-_Neubauer_Chamber_Cell_Counting-_1_Oscar_Bastidas. (accessed on 12 March2020).
4. Schneider, C.A.; Rasband, W.S.; Eliceiri, K.W. NIH Image to ImageJ: 25 years of image analysis. *Nat. Methods* **2012**, *9*, 671–675.
